# Skeletal muscle adaptation to indirect electrical stimulation: divergence between microvascular and metabolic adaptations

**DOI:** 10.1113/EP091134

**Published:** 2023-04-07

**Authors:** Roger W. P. Kissane, David Hauton, Peter G. Tickle, Stuart Egginton

**Affiliations:** ^1^ Department of Musculoskeletal & Ageing Science, Faculty of Health & Life Sciences University of Liverpool Liverpool UK; ^2^ School of Biomedical Sciences, Faculty of Biosciences University of Leeds Leeds UK; ^3^ Metabolomics Research Group, Department of Chemistry University of Oxford Oxford UK

**Keywords:** angiogenesis, exercise, metabolomics, muscle stimulation

## Abstract

Exercise involves a complex interaction of factors influencing muscle performance, where variations in recruitment pattern (e.g., endurance vs. resistance training) may differentially modulate the local tissue environment (i.e., oxygenation, blood flow, fuel utilization). These exercise stimuli are potent drivers of vascular and metabolic change. However, their relative contribution to adaptive remodelling of skeletal muscle and subsequent performance is unclear. Using implantable devices, indirect electrical stimulation (ES) of locomotor muscles of rat at different pacing frequencies (4, 10 and 40 Hz) was used to differentially recruit hindlimb blood flow and modulate fuel utilization. After 7 days, ES promoted significant remodelling of microvascular composition, increasing capillary density in the cortex of the tibialis anterior by 73%, 110% and 55% for the 4 Hz, 10 and 40 Hz groups, respectively. Additionally, there was remodelling of the whole muscle metabolome, including significantly elevated amino acid turnover, with muscle kynurenic acid levels doubled by pacing at 10 Hz (*P* < 0.05). Interestingly, the fatigue index of skeletal muscle was only significantly elevated in 10 Hz (58% increase) and 40 Hz (73% increase) ES groups, apparently linked to improved capillary distribution. These data demonstrate that manipulation of muscle recruitment pattern may be used to differentially expand the capillary network prior to altering the metabolome, emphasising the importance of local capillary supply in promoting exercise tolerance.

## Abstract (highlights)

- **What is the central question of this study?**
  Can we manipulate muscle recruitment to differentially enhance skeletal muscle fatigue resistance?
- **What is the main finding and its importance?**
  Through manipulation of muscle activation patterns, it is possible to promote distinct microvascular growth. Enhancement of fatigue resistance is closely associated with the distribution of the capillaries within the muscle, not necessarily with quantity. Additionally, at the acute stages of remodelling in response to indirect electrical stimulation, the improvement in fatigue resistance appears to be primarily driven by vascular remodelling, with metabolic adaptation of secondary importance.

## 1 INTRODUCTION

Skeletal muscle is highly adaptable, undergoing dynamic changes in mass, vascular supply and metabolic substrate use in response to different environmental stimuli. For example, the response to prolonged rest or immobilization includes muscle atrophy and capillary rarefaction (Al‐Shammari et al., 2019; Graham et al., 2019), while increased activity (through exercise training) leads to muscle hypertrophy, increased capillarization and altered metabolism (Hoier et al., 2013; Mortensen et al., 2019; Morville et al., 2020). Understanding the holistic response to altered activity patterns is a prerequisite to developing targeted therapeutic exercise interventions for specific pathological conditions (Sanford et al., 2020).

Chronic indirect electrical stimulation (ES) may be used to manipulate the local environment of muscle fibres (Hargreaves et al., 1990), driving adaptive remodelling of oxygen supply through expansion of the microvascular bed (Pette et al., 1973) and intermediary metabolism (Brownson et al., 1988). In skeletal muscle of rodents, ES is a potent modulator of key angiogenic proteins (Egginton, 2009), with low frequency (10 Hz) stimulation of the peroneal nerve elevating vascular endothelial growth factor (VEGF) in the extensor digitorum longus (EDL) and tibialis anterior (TA) (Annex et al., 1998), and subsequently increasing capillary supply (Linderman et al., 2000). Similar low frequency (<10 Hz) ES significantly elevated hexokinase, citrate synthase and succinate dehydrogenase enzyme activity (Pette et al., 1973). Comparable stimulation regimes increased carbonic anhydrase activity and actin expression, with myosin heavy chain and glyceraldehyde 3‐phosphate dehydrogenase showing transiently increased expression (Brownson et al., 1988). Furthermore, continuous stimulation of TA muscle at 10 Hz increased the concentration of cyclic AMP, adrenergic receptor expression and adenylate cyclase activity (Kraus et al., 1992). These complementary changes are thought to underlie improved fatigue resistance. By contrast, one study found no effect of ES on oxidative capacity of skeletal muscle (Goldspink et al., 1991).

While ES is known to exert a potent influence on muscle phenotype, the magnitude and time course of changes in the microvascular supply and enzyme activity varies with stimulation frequency. Stimulation at 10 and 40 Hz both elicit a significant increase in limb blood flow and oxygen consumption (Hudlicka et al., 1984), but the temporal angiogenic response differs greatly. Hudlická and Tyler (1984) showed that stimulation at 40 Hz rapidly expand the capillary network with 10 Hz presenting a delayed response, while a comparable study (Hudlická et al., 1988) suggested the opposite temporal response. Low frequency stimulation (10 Hz) preferentially increases succinate dehydrogenase (SDH) activity in Type IIa fibres, peaking at 7 days with a delayed increase in Type IIb occurring at 14 days. By contrast, high frequency (40 Hz) stimulation rapidly increased SDH activity of both Type IIa and b after just 7 days (Pette & Tyler, 1983).

Depending on the basal phenotype of skeletal muscle activated, it is possible to drive differential adaptive remodelling responses by ES (Hudlická et al., 1988), and through manipulation of stimulation frequency there is the potential to develop targeted therapeutic paradigms optimized for different pathologies. Low voltage ES of ischaemic muscle has been shown to increase expression of both VEGF and hepatocyte growth factor protein levels, demonstrating a capacity to increase pro‐angiogenic signals in situ (Nagasaka et al., 2006) and overcome the microvascular deficit (Deveci & Egginton, 2002). Similarly, following spinal cord injury rats reduced oxidative fibre content but increased glycolytic fibre content in TA, while ES increased oxidative fibre size, ATPase activity, SDH activity and capillary supply (Martin et al., 1992). Also, ES of TA in paraplegic rats led to increased expression of monocarboxylate transporters (MCT) in glycolytic and oxidative fibres, with a greater efflux of lactate from stimulated muscle in glycolytic fibres, while increased lactate uptake was correlated with both MCT expression and lactate dehydrogenase activity (McCullagh et al., 1997). These observations indicate that ES has the potential to preserve oxidative capacity of muscle by altering expression of metabolic enzymes and substrate carrier proteins.

Previous work has focused primarily on a restricted number of enzymatic targets which provides only a snapshot of information, whereas a comprehensive metabolomics approach provides insight into modulation of metabolic pathways. The principal objective of this study was therefore to determine how ES affects adaptive remodelling of rat skeletal muscle structure and underlying metabolome through the use of distinct ES parameters to manipulate pertinent environmental factors (e.g., muscle blood flow, force production and fuel utilization) (Hawker & Egginton, 1999). We hypothesized that pacing of skeletal muscle would drive stimulation frequency‐dependent angiogenic remodelling and complementary metabolic changes (e.g., metabolites associated with the tricarboxylic acid (TCA) cycle and acylcarnitine activity), driven by transient changes in tissue oxygen availability, that facilitate increased oxidative capacity. Exploiting implantable stimulators we quantified changes in global and local microvascular supply to the TA muscle, and used novel oxygen transport modelling techniques to predict the functional consequence of structural remodelling (Al‐Shammari et al., 2019). In addition, we utilized a multi‐platform approach using liquid chromatography–mass spectrometry to identify metabolites of the central energy metabolism pathways to determine the impact of ES on skeletal muscle metabolic adaptation.

## 2 METHODS

### 2.1 Ethical approval

**FIGURE 1:**
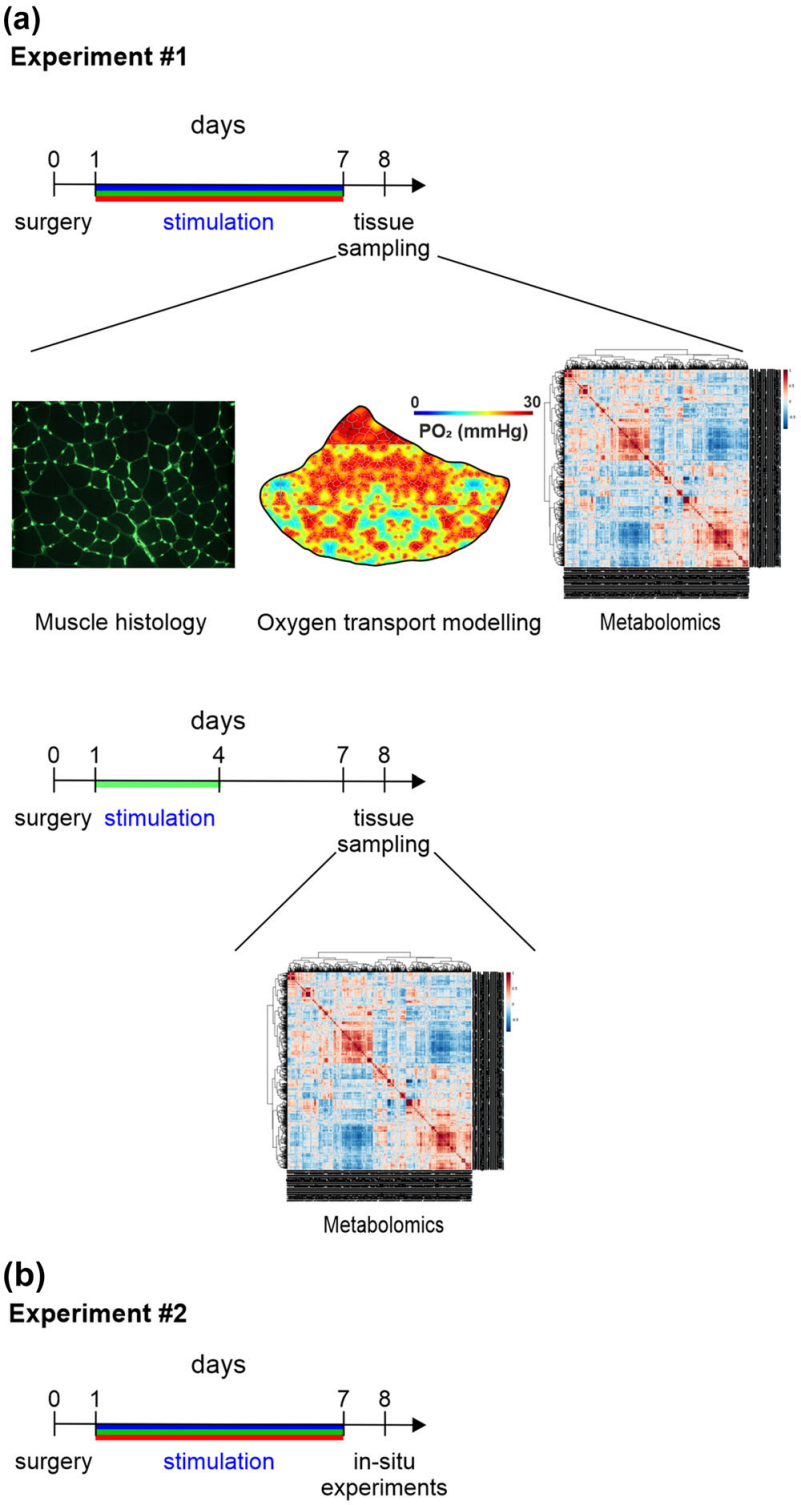
Experimental design and methods.

All animal work was approved by the University of Leeds Animal and Welfare Ethical Review Board and carried out in accordance with the Animals (Scientific Procedures) Act 1986 (under Home Office Project licence: 70/08674). This work conforms to the ethical requirements outlined by the *Experimental Physiology* in accordance with guidelines for animal work (Grundy, 2015; Percie du Sert et al., 2020). Twelve‐week‐old male Wistar rats (sourced from the University of Leeds colony) were housed in groups of three or four on a 12‐h light‐dark cycle at 21°C with ad libitum access to food and water, and 50 animals (body mass 248 ± 2.4 g) were split across two experimental conditions (Figure 1, Table 1). Briefly, all animals underwent the same 7‐day experimental procedures, with varying terminal experimentation. Experiment 1 animals were culled through a Schedule 1 method (cerebral concussion and cervical dislocation) and the TA immediately snap‐frozen for histological and metabolic profiling (Figure 1a). Animals in Experiment 2 underwent terminal anaesthesia for in situ characterization of EDL blood flow and muscle performance (Figure 1b).

**TABLE 1:** Animal morphometrics.

Experiment 1	Body weight (g)	Relative TA weight (% body weight)
Control	231.8 ± 3.4 (6)	0.182 ± 0.007 (6)
4 Hz	247.9 ± 2.0 (7)	0.179 ± 0.005 (7)
10 Hz_(3)_	250.8 ± 5.0 (5)	0.174 ± 0.007 (5)
10 Hz	245.8 ± 6.9 (5)	0.174 ± 0.006 (5)
40 Hz	237.5 ± 8.3 (6)	0.180 ± 0.009 (6)

Experiment 2		Relative EDL weight (% body weight)
Control	260.6 ± 10.7 (5)	0.064 ± 0.002 (5)
4 Hz	250.8 ± 5.8 (5)	0.051 ± 0.003 (5)
10 Hz	260.0 ± 7.2 (6)	0.054 ± 0.003 (6)
40 Hz	253.0 ± 7.5 (5)	0.051 ± 0.002 (5)

Values in parentheses are sample size, *n*.

### 2.2 Surgical procedures

Animal surgery was conducted under aseptic conditions with anaesthesia induced using 5% isoflurane (IsoFlo, 2 litres min^−1^ O_2_ flow), and the surgical plane maintained with 2.5% isoflurane (2 litres min^−1^ O_2_ flow). Following confirmation of anaesthetic plane through absent blink and toe–hindlimb reflex, surgery could begin. Animals underwent indirect ES of the hindlimb ankle flexors via the deep lateral peroneal nerve. Miniaturized, battery‐powered electrical stimulators were coated with hypo‐allergenic beeswax and placed in a subcutaneous pouch on the mid‐thoracic region of the back (Linderman et al., 2000). The stimulator was inserted through a 2 cm incision and sutured into place. Stimulating electrodes made from Teflon coated seven‐strand braided stainless steel wires (A‐M Systems, Sequim, WA, USA) were tunnelled distally towards the right hindlimb under the skin, and a loose wire loop was sutured into place near the hip to minimize tension in the wire during locomotion. Electrodes were passed through the vastus lateralis using a 23 g needle towards the peroneal nerve. Once in place, covering fascia across the superficial muscles were closed with absorbable 6‐0 suture and the skin closed with braided 4‐0 silk suture (Ethicon/Johnson & Johnson Medical, New Brunswick, NJ, USA). Animals received s.c. injections of analgesic (30 μg kg^−1^ buprenorphine; Vetergesic, Ceva Animal Health Ltd, Amersham, UK) and antibiotic (2.5 mg kg^−1^ enrofloxacin; Baytril, Bayer, Reading, UK) immediately after surgery, repeated for 2 days post‐surgery. The animals were given 24 h to recover before the stimulators were activated, allowing sufficient time for surgical trauma/inflammation to subside as well as normal locomotion to resume.

### 2.3 Stimulation protocol

**FIGURE 2:**
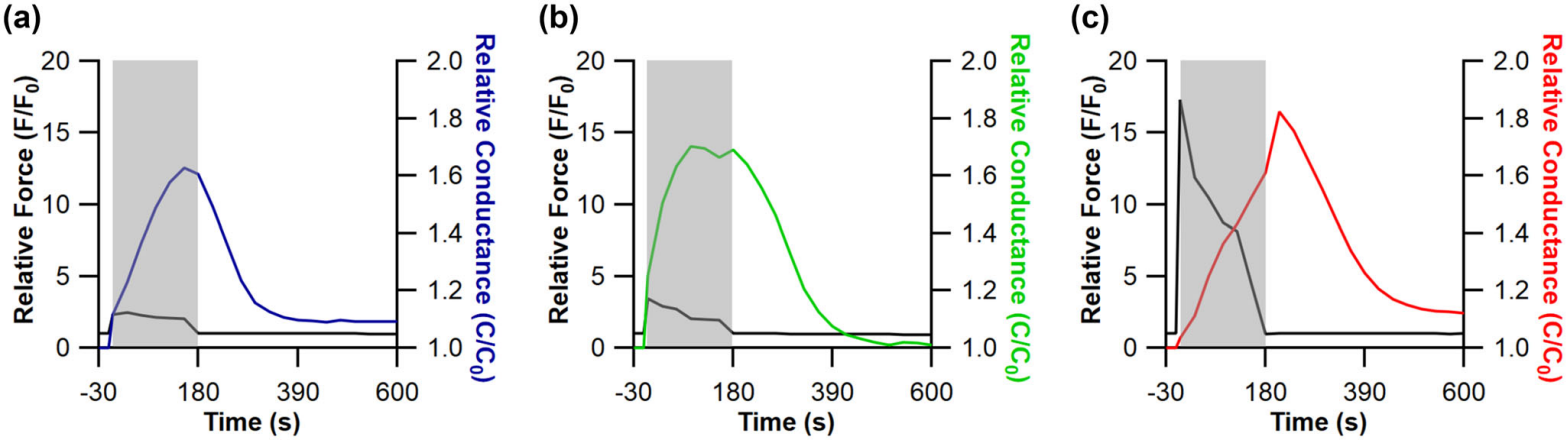
Blow flow and force kinetics of the extensor digitorum longus to indirect electrical stimulation. Indirect electrical stimulation was delivered at 4 Hz (a), 10 Hz (b), and 40 Hz (c) for 3 min (grey shaded region) to the extensor digitorum longus of male Wistar rats (*n* = 4). Functional hyperaemia is evident through changes in relative conductance (coloured lines) within the shaded stimulation period. Progressive increase in conductance reaches a peak around 3 min with 4 Hz, is reached earlier with increased activity at 10 Hz, but is delayed until after stimulation at 40 Hz due to attenuation of arterial stenosis as muscle fatigue develops. Conductance returns towards baseline values in the successive white region post‐stimulation, with a likely contribution from reactive hyperaemia delaying this at 40 Hz.

Indirect ES was delivered using a supramaximal voltage, delivered at pre‐set frequencies of either 4 Hz or 10 Hz continuously for 8 h, or 40 Hz for 8 h with 15 min on–45 min off, for 7 days. Stimulation protocols were executed during the dark cycle (i.e., when the animals were asleep). Stimulation duration was controlled rather than absolute number of stimuli received, owing to the supra‐physiological effects of 20 h of stimulation required for the 4 Hz protocol to match dosage of 10 Hz (unpublished data show a pathophysiological fibre atrophy after 7 days of 20 h stimulation). Through manipulation of stimulation frequency, we were able to modulate the level of force recruitment, active‐ and reactive‐hyperaemia (Figure 2), known potent drivers of microvascular and metabolic remodelling. To discern the temporal nature of the metabolic remodelling we also completed an additional experimental group of 10 Hz stimulation for only 3 days (10 Hz_(3)_) (Figure 1a).

### 2.4 Experiment 1

Whole TA muscles were carefully dissected from the hindlimb and weighed. Muscles were split into three equal portions with the proximal and distal portions frozen in liquid nitrogen for metabolomics analysis, while the mid portions were snap‐frozen in liquid nitrogen‐cooled isopentane for histological sampling. All tissue was stored at −80°C until further use.

#### 2.4.1 Histological analysis

**FIGURE 3:**
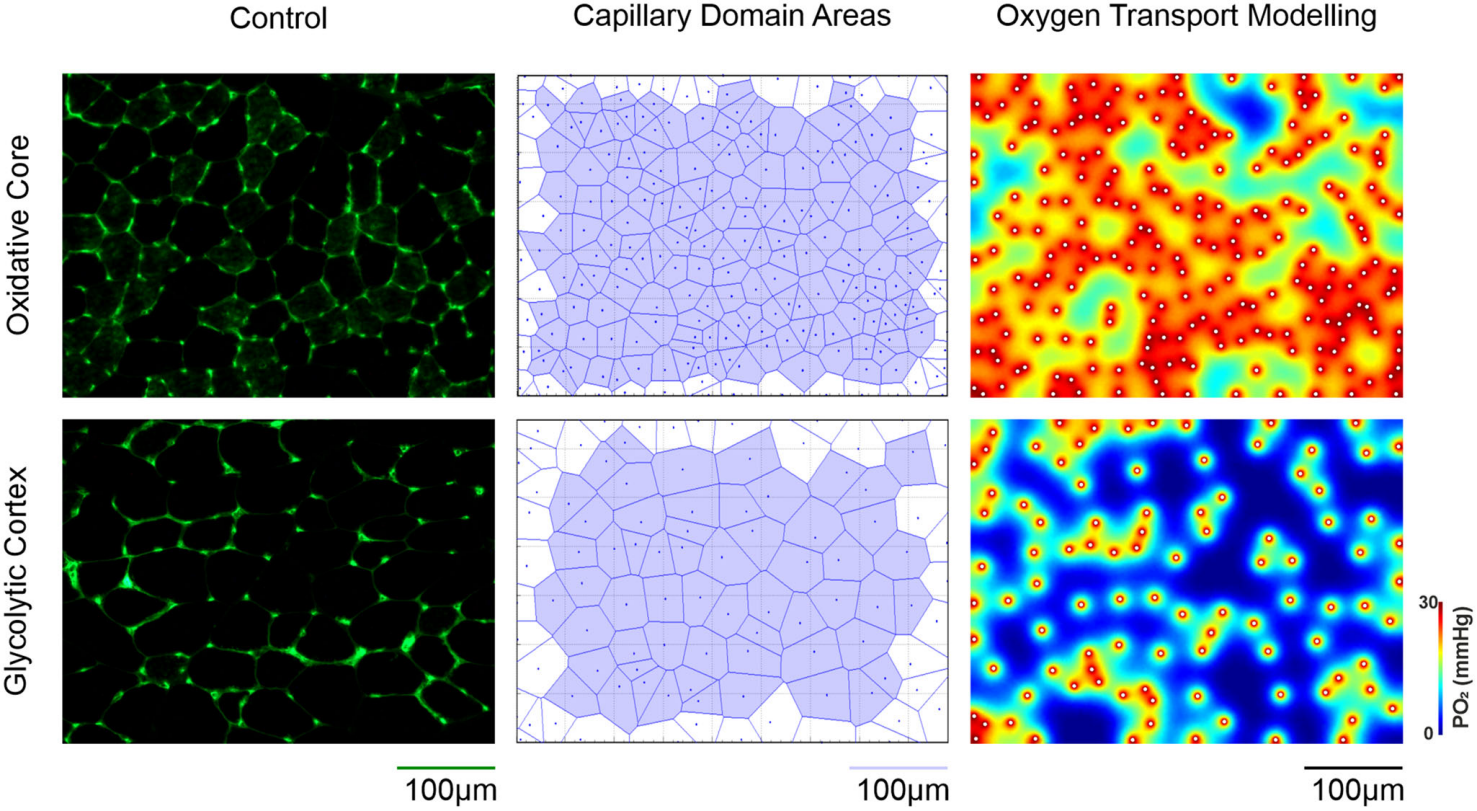
Microvascular distribution throughout the tibialis anterior. Example histological images from the oxidative core and glycolytic cortex labelled with *Griffonia simplicifolia* lectin‐1. Corresponding capillary domain area derived from the histological images highlight the difference in supply area between the core and cortex. Finally, the consequence of capillary domain area on estimates of tissue partial pressure of oxygen ($P_{O_{2}}$).

**FIGURE 4:**
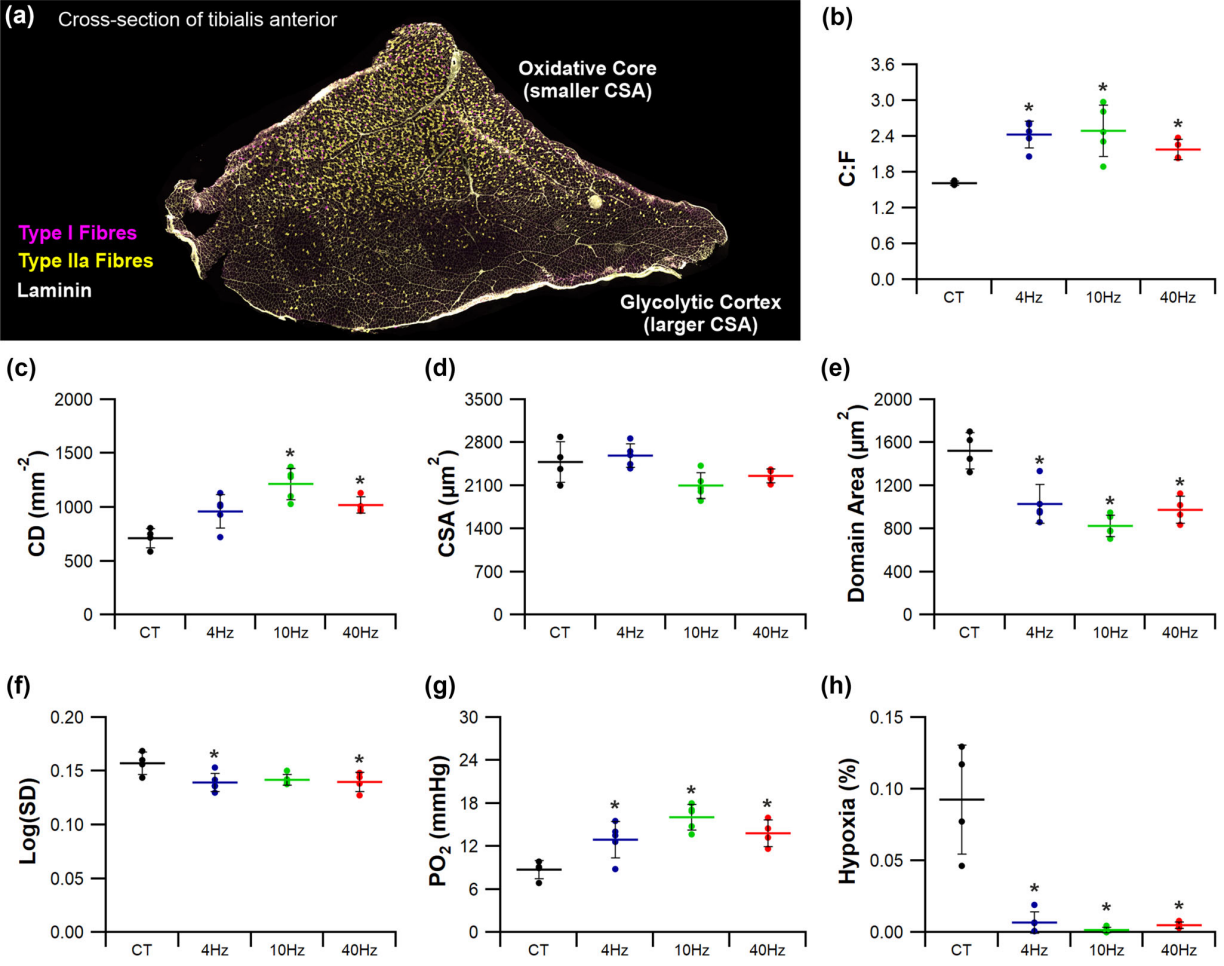
Whole tibialis anterior response to indirect electrical stimulation. (a) The tibialis anterior is a heterogeneous muscle comprising a deep oxidative core and a superficial glycolytic cortex. Immunohistochemical staining of Type I fibres (magenta), Type IIa (yellow), Type IIb/x (unstained) and laminin (white). (b,c) ES significantly enhanced capillary‐to‐fibre ratio (b) with 10 and 40 Hz significantly increasing capillary density (c). (d) There was no significant effect of ES on fibre cross‐sectional‐area. (e,f) ES significantly reduced the capillary domain area (e), with 4 and 40 Hz significantly reducing capillary heterogeneity (f). (g,h) Consequently, modelled tissue $P_{O_{2}}$ (g) was significantly improved across all three stimulation frequencies, as was the modelled extent of relative tissue hypoxia (h). Data derived from 5 regions of interest across the whole TA. Means ± SD, *P < 0.05 vs. control tissue (CT). (b–h) CT (*n* = 4), 4 Hz (*n* = 5), 10 Hz (*n* = 5) and 40 Hz (*n* = 4).

Serial cryostat (−20°C) sections (10 μm) were attached to polysine adhesion slides (VWR international), then fixed with 2% paraformaldehyde for 2 min. Following 10 washes in phosphate‐buffered saline (PBS), sections were stained with *Griffonia simplicifolia* lectin‐1 (Vector Laboratories, Peterborough, UK; diluted 1:200 with PBS) to identify capillaries through its affinity to proteoglycans in the glycocalyx. Slides were incubated for 1 h, then washed and mounted in VectaShield (Vector, H‐1400). Five regions of interest (0.145 mm^2^) were imaged, spanning two metabolically distinct regions within the TA: the oxidative core (×2 fields) and the glycolytic cortex (×3 fields) (Figure 3) (Kissane et al., 2019). Capillary‐to‐fibre ratio (C:F), capillary density (CD) and mean fibre cross‐sectional area (CSA) were derived from histological cross‐sections using an unbiased sampling regime. These commonly used global indices describe gross changes in capillary supply, but lack resolution for local spatial distribution in capillarity (Egginton & Gaffney, 2010; Kissane et al., 2021; Olfert et al., 2016). Therefore, we present further local capillary indices of tissue supply area (capillary domain area; CDA) and heterogeneity (standard deviation of the log‐transformed CDA; logSD). Additionally, tissue oxygen transport kinetics were modelled on histologically derived capillary distributions using capillary domain boundaries as geometric constraints (trapping regions) to estimate tissue partial pressure of oxygen ($P_{O_{2}}$) at rest, and a simulated high tissue oxygen consumption rate representative of intense exercise (Al‐Shammari et al., 2019; Hauton et al., 2015). One potential shortcoming of this approach is tissue‐wide coverage of metabolomics analysis whilst the histological analysis possessed more regional focus to highlight any differential effects due to fibre‐type specific phenotype, but subdivision of muscle samples to discretely analyse the metabolome of each histological sampling region would yield insufficient tissue mass. Therefore, we also provide histological analysis normalising the relative indices from the core:cortex, to facilitate comparison with observations for metabolomics analysis (whole TA data presented in Figure 4).

#### 2.4.2 Metabolomic analysis

##### 2.4.2.1 Extraction protocol

Tissue samples were homogenized (final concentration 100 mg ml^−1^) in extraction buffer (80% methanol in deionized water) using an Ultraturrax blade homogenizer (IKA England Ltd, Oxford, UK). Samples were centrifuged and the supernatant recovered, passed through 10 kDa molecular mass ‘cut‐off’ filters (Millipore, Burlington, MA, USA) and double‐stranded DNA concentration estimated in the resulting fraction (Nanodrop Spectrophotometer, Mettler‐Toledo, Columbus, OH, USA). All tissue samples were diluted and normalized with respect to DNA concentration.

##### 2.4.2.2 Anion exchange analysis

Ion exchange chromatography was performed using an ICS‐5000+ HPLC system (Thermo Fisher Scientific, Waltham, MA, USA) incorporating an electrolytic anion generator (KOH) which was programmed to produce a OH^–^ gradient over 37 min. An inline electrolytic suppressor removed OH^–^ ions and cations from the post‐column eluent stream prior to mass spectrometry (MS) analysis (Thermo Scientific Dionex AERS 500). A 10 μl partial loop injection was used for all analyses and the chromatographic separation was performed using a Thermo Scientific Dionex IonPac AS11‐HC 2 × 250 mm, 4 μm particle size column with a Dionex Ionpac AG11‐HC 4 μm 2 × 50 guard column inline and an IC flow rate of 0.250 ml min^−1^. The total run time was 37 min and the hydroxide ion gradient comprised the following: 0 min, 0 mM; 15 min, 60 mM; 25 min, 100 mM; 30 min, 100 mM; 30.1 min, 0 mM. Analysis was performed in negative ion mode using a scan‐range from *m/z* 60 to 900 and resolution set to 70,000. The tune file source parameters were set as follows: sheath gas flow 60 ml min^−1^; auxiliary gas flow 20 ml min^−1^; spray voltage 3.6 V; capillary temperature 320^o^C; S‐lens Radio frequency (RF) value 70; heater temperature 350°C. Automatic gain control (AGC) target was set to 1 × 10^6^ ions and the max Incidence time (IT) value was 250 ms. The column temperature was kept at 30°C throughout the experiment. Full scan data were acquired in continuum mode.

##### 2.4.2.3 C18 reversed phase (underivatized)

C18 reversed‐phase analysis of underivatized samples was performed using a Thermo Utimate 3000 UHPLC system (Thermo Fisher Scientific) with a gradient elution programme coupled directly to a Q‐Exactive HF Hybrid Quadrupole‐Orbitrap mass spectrometer (Thermo Fisher Scientific). A 5 μl partial loop injection was used for all analyses with pre and post injection wash programme. A Waters (Milford, MA, USA) CORTECS UPLC T3 1.6 μm (2.1 × 100 mm) column (maintained at 40°C) was used with a flow rate of 0.4 ml min^−1^; total run time was 18 min. Mobile phase A comprised milli‐Q water with 0.1% formic acid, and mobile phase B was 100% methanol with 0.1% formic acid. The gradient elution programme was as follows: 0 min, 5% B; 4 min, 50% B; 12 min, 99% B; 15 min, 99% B; 15.1 min, 5% B. Mass spectrometry analysis was performed in positive and negative ion mode separately using a scan‐range from *m/z* 60 to 900 and resolution set to 70,000. The tune file source parameters were set as follows: sheath gas flow 60 ml min^−1^; auxiliary gas flow 20 ml min^−1^; spray voltage 3.6 V; capillary temperature 320^o^C; S‐lens RF value 70; heater temperature 350°C. Full MS setting were AGC target 5 × 10^6^ ions and the max IT value was 120 ms. Full scan data were acquired in continuum mode. A data directed tandem mass spectrometry (ddMS^2^) method was utilized with no inclusion list. The orbitrap detector and higher‐energy collisional dissociation (HCD) setting for ddMS^2^ were as follows: microscans 2, resolution 17,500, AGC target 5 × 10^4^ ions, maximum IT 80 ms, loop count 10 and normalized collision energy (NCE) 35.

##### 2.4.2.4 C18 reversed phase (derivatized)

The liquid chromatography (LC)–MS method used a sample derivatization protocol to label primary and secondary amines followed by analysis based on a modified version of the Waters AccQ‐Tag method (Salazar et al., 2012). C18 reversed‐phase analysis of derivatized samples was also performed using the Thermo Utimate 3000 UHPLC system coupled directly to a Q‐Exactive HF Hybrid Quadrupole‐Orbitrap mass spectrometer. A 5 μl partial loop injection was used for all analyses with pre‐ and post‐injection wash programme. A Waters AccQ‐Tag column (2.1 × 100 mm, maintained at 40°C) was used with a flow rate of 0.5 ml min^−1^; total run time was 9.5 min. Mobile phase A and B comprised commercially available AccQ‐Tag reagents prepared as recommended by Waters. The gradient elution programme was modified from the published AccQ‐Tag method as follows: 0 min, 0.1% B; 0.54 min, 9.1% B; 5.74 min, 21.2% B; 7.74 min, 59.6% B; 8.04 min, 90% B; 8.05 min, 90% B; 8.64 min, 0% B; 9.5 min, 0.1% B. MS analysis was performed in positive ion mode separately using a scan‐range from *m*/*z* 70 to 1050 and resolution set to 70,000. The tune file source parameters were set as follows: sheath gas flow 60 ml min^−1^; auxilary gas flow 20 ml min^−1^; spray voltage 3.6v; capillary temperature 320°C; S‐lens RF value 70; heater temperature 350°C. Full MS setting were AGC target 3 × 10^6^ ions and the max IT value was 200 ms. Full scan data were acquired in continuum mode.

##### 2.4.2.5 Hydrophobic interaction liquid chromatography–MS

Acyl‐carnitines were separated and resolved using hydrophobic‐interaction liquid chromatography–mass spectrometry (HILIC‐MS). Briefly, samples were eluted using a binary solvent acetonitrile:water (50%:50%) containing ammonium acetate (10 mM final concentration (solvent A)) and acetonitrile:water (95%:5%) containing ammonium acetate (10 mM final concentration (solvent B)). Samples were resolved using a linear gradient (10 min: 100% solvent A to 100% solvent B) and re‐equilibrated with 100% solvent A. Putative compounds were identified with reference to authenticated standards for selected acyl‐carnitines using retention time, accurate mass and fragmentation pattern to identify individual compounds. Concentrations calculated with reference to specific standard curves.

##### 2.4.2.6 Data processing

Ion species were identified with reference to an ‘in‐house’ database created from authenticated standards. Briefly, pure compounds were purchased from chemical suppliers (e.g., Merck Life Science UK Ltd, Gillingham, UK; Tocris Bioscience, Bristol, UK; Tokyo Chemicals Industry, Oxford, UK). These standards were then diluted in solvent (80% methanol) and separated chromatographically by different methods. Each compound was then examined using a Q Exactive mass spectrometer (Thermo Fisher Scientific) and the authenticated standard identified by collection of discrete data: this included chromatographic retention time, accurate mass (five decimal places) and compound fragmentation, thus allowing identification of isomers with reference to differing fragmentation and retention characteristics.

Raw data files were processed using Progenesis QI (Waters). This process included alignment of retention times, picking by identification of natural abundance isotope peaks, characterising multiple adduct forms and identification of metabolites using our in‐house database. Retention times, accurate mass values, relative isotope abundances and fragmentation patterns were compared between authentic standards and the samples measured. Identifications were accepted only when the following criteria were met: <5 ppm differences between measured and theoretical mass (based on chemical formula); <30 s differences between authenticated standard and analyte retention times; isotope peak abundance measurements for analytes were >90% matched to the theoretical value generated from the chemical formula. Where measured, fragmentation patterns were matched to least the base peak and two additional peak matches in the MS/MS spectrum to within 12 ppm.

### 2.5 Experiment 2

#### 2.5.1 in situ muscle function

Anaesthesia was induced with isoflurane (4% in O_2_) and maintained by constant infusion (30–35 mg kg^−1^ h^−1^) of alfaxalone (Alfaxan, Jurox, Crawley, UK) through an implanted catheter in the descending jugular vein (Tickle et al., 2020). An implanted catheter in the carotid artery was connected to a pressure transducer (ADInstruments, Oxford, UK) to monitor blood pressure and heart rate throughout the protocol, while spontaneous breathing was facilitated by a tracheotomy. The TA was then carefully removed without damage to the underlying EDL and snap‐frozen for subsequent analyses. The distal EDL tendon was attached to an ergometer lever arm (305B, Aurora Scientific Inc., Aurora, Ontario, Canada) to enable quantification of isometric contractile forces. The peroneal nerve was exposed and stimulated using stainless steel electrodes sutured parallel to the neural sheath. Optimal muscle length and electrical current delivery for maximal force generating capacity were determined prior to fatigue assessment. Muscle fatigability was assessed through the application of a 10 Hz stimulation protocol (pulse width 0.3 ms) for 3 min. The fatigue index (FI) was calculated as final tension/peak tension × 100, using five consecutive twitches in each case. Perivascular flow probes (0.7PSB, Transonic, Ithaca, NY, USA) were placed around the proximal femoral artery to measure the rate (ml min^−1^) of arterial blood flow. An effect of pacing upon arterial blood flow was assessed by comparison of functional hyperaemia (peak/resting blood flow) during fatigue stimulation. The EDL is often used in conjunction with, or as a proxy to, the tibialis anterior to assess muscle function (Hudlicka et al., 1994; Kwong & Vrbova, 1981) owing to a similar phenotype and motor function. This is considered appropriate within the context of this study as induced contractile activity of the TA would alter its metabolomic profile, rendering the muscle unusable. The TA and EDL both show identical temporal angiogenic structural changes in response to hypoxia (Deveci et al., 2002) and indirect ES (Brown et al., 1995) with comparable fatigue responses (Grasa et al., 2014). Consequently, any link between metabolic, structural and functional changes is expected to be translatable across the two experiments. Following completion of fatigue experimentation, animals were culled by cervical dislocation.

### 2.6 Statistical analysis

Differentiation in structural indices is statistically viable with a minimum of four biological replicates (Egginton, 1988), which was achieved across all experimental groups, with data analysed using a one‐way analysis of variance (ANOVA) with Tukey's *post hoc* test in SPSS Statistics (v.25; IBM Corp., Armonk, NY, USA). The analytical robustness of our structural data analysis is further improved using local capillary indices and mathematical modelling (Egginton, 1990; Kissane et al., 2021). Metabolomic comparison between the treatment and control samples were analysed using univariate statistical analysis determining fold‐change, Student's *t*‐test between experimental groups for compound features, and combined in volcano plots (false discovery rate (FDR)‐adjusted *P*‐values). Principal component analysis (PCA) and partial least‐squares discriminant analysis (PLS‐DA) were also used to analyse the patterns in metabolomic profiles among treatments. All data are presented as means ± SD. Statistical significance was assigned at *P* ≤ 0.05.

## 3 RESULTS

### 3.1 Histological analysis

**FIGURE 5:**
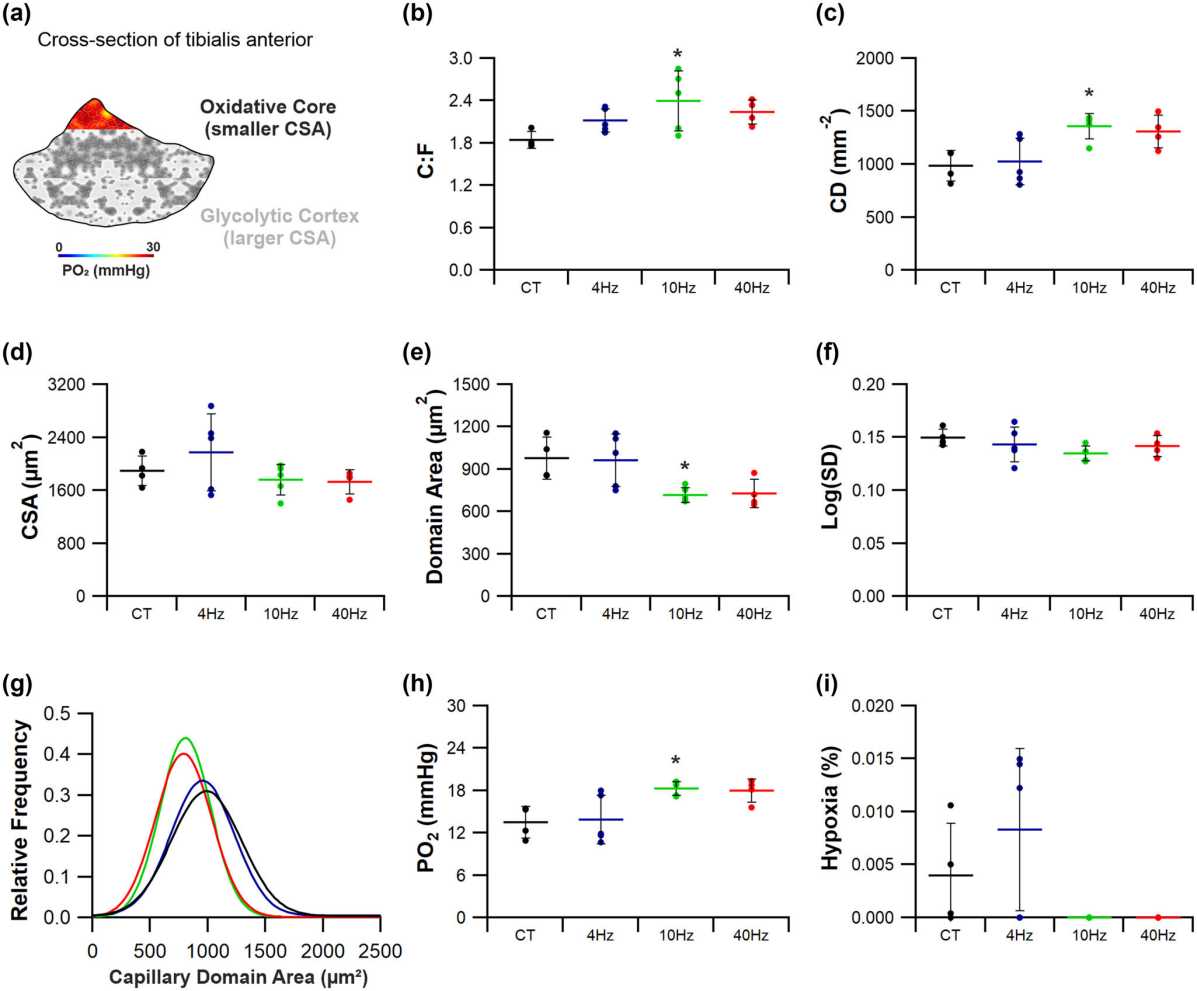
Angiogenic response in the tibialis anterior core to indirect electrical stimulation. (a) The posterior/medial compartment of TA is highly oxidative in phenotype. (b,c) Only 10 Hz ES significantly elevated capillary‐to‐fibre ratio (b) and capillary density (c). (d) Muscle fibre cross‐sectional‐area was unchanged across all stimulation groups. (e–g) Capillary domain area (e) appeared to respond greatest to the higher frequency stimulation, while there was no change in capillary heterogeneity (f) despite a leftward shift in capillary domain distribution (g) for the 10 and 40 Hz ES groups. (h,i) Finally, only 10 Hz stimulation significantly elevated modelled tissue $P_{O_{2}}$ (h), while there were no significant changes to the estimates of tissue hypoxia (i). Means ± SD, *P < 0.05. Control tissue (CT) (*n* = 4), 4 Hz (*n* = 5), 10 Hz (*n* = 5) and 40 Hz (*n* = 4).

**FIGURE 6:**
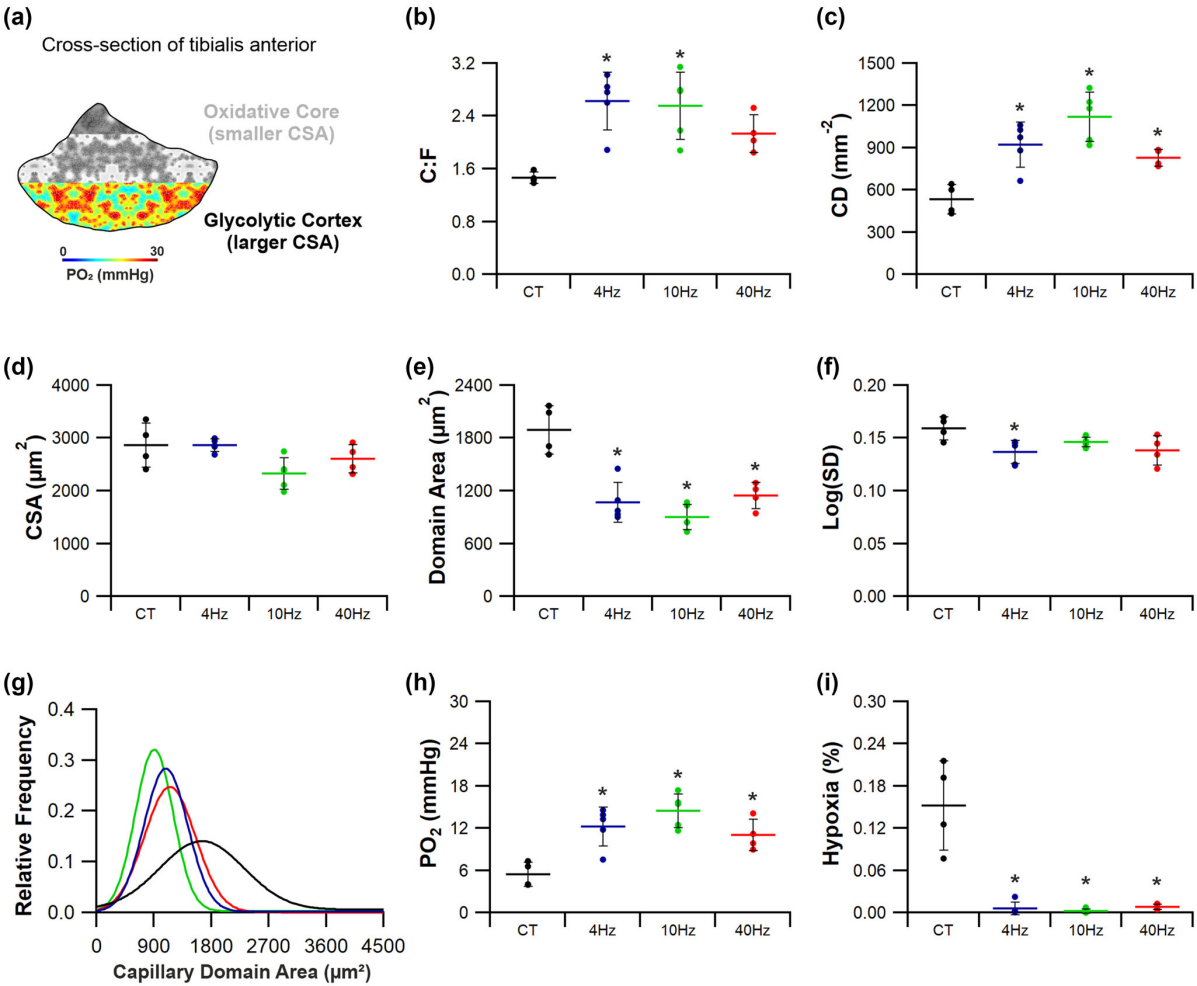
Angiogenic response in the tibialis anterior cortex to indirect electrical stimulation. (a) The anterior/lateral compartment of the tibialis anterior is highly glycolytic in phenotype. (b–d) All three stimulation frequencies significantly enhanced microvascular composition in the TA cortex, with changes in capillary‐to‐fibre ratio (b) and capillary density (c) unrelated to changes in fibre cross‐sectional‐area (d). (e,f) Capillary domain area (e) was significantly decreased across all three ES paradigms, with capillary heterogeneity (f) only significantly reduced in the 4 Hz group. (g) This enhancement in microvascular supply led to a leftward shift in capillary domain distribution across all three groups. (h,i) Subsequently modelled tissue $P_{O_{2}}$ (h) was significantly enhanced across all three groups, with a significant decrease in modelled tissue hypoxia (i). Means ± SD, *P < 0.05. Control tissue (CT) (*n* = 4), 4 Hz (*n* = 5), 10 Hz (*n* = 5) and 40 Hz (*n* = 4).

**FIGURE 7:**
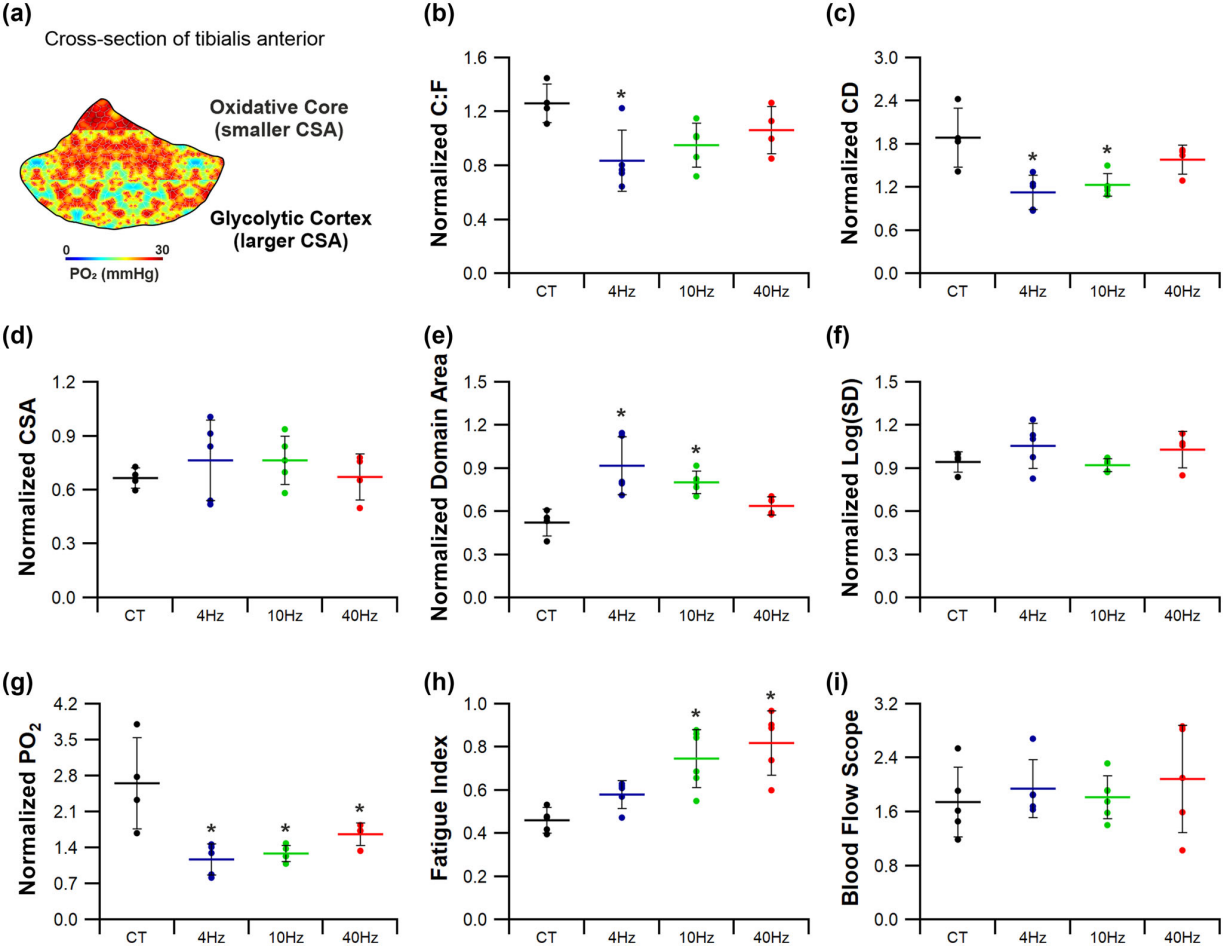
Differential response of the tibialis anterior. (a) Data presented as tibialis core relative to the cortex to highlight the overall shift in phenotype of the muscle. (b,c) 4 Hz stimulation significantly altered the relative capillary‐to‐fibre ratio (b) and capillary density (c) of the TA, with the cortex becoming more similar in morphology to that of the core. (d) All three stimulation regimes had no significant effect on muscle fibre cross‐sectional‐area. (e)The significant shift in capillary density seen with 4 and 10 Hz is echoed by the significant increase in normalized capillary domain area. (f) There was, however, no significant change in normalized capillary heterogeneity. (g) The significant decrease in normalized modelled tissue $P_{O_{2}}$ suggests a more pronounced improvement in modelled cortex $P_{O_{2}}$ confirmed in Figure 2. (h,i) There was a significant enhancement in extensor digitorum longus fatigue resistance (h) following 10 and 40 Hz ES, which is independent of changes in hindlimb blood flow (i). Means ± SD, *P < 0.05. (a–g) Control tissue (CT) (*n* = 4), 4 Hz (*n* = 5), 10 Hz (*n* = 5) and 40 Hz (*n* = 4); (h,i) CT (*n* = 5), 4 Hz (*n* = 5), 10 Hz (*n* = 6), and 40 Hz (*n* = 5).

Indirect ES was a potent stimulus for structural adaptive remodelling across the TA (Figures 4, 5, 6, 7). While changes across the whole muscle may provide initial evidence for adaptive remodelling, it is more prudent to consider the two phenotypically extreme compartments of the TA, given the inherent heterogeneity in fibre type composition (Figure 4a). Therefore, evidence for remodelling is presented in the oxidative core (Figure 5) and the glycolytic cortex (Figure 6). In order to discern shifts in gross phenotype, the ratio of indices for the core:cortex is used to illustrate shifts across the whole muscle (Figure 7).

#### 3.1.1 Tibialis anterior oxidative core

Within the oxidative core (Figure 5a), significantly increased capillarity was found only in response to pacing at 10 Hz (C:F = 2.40 ± 0.42, *P =* 0.032; CD = 1360 ± 122 mm^−2^, *P =* 0.021; Figure 5b,c) when compared to control (C:F = 1.84 ± 0.12 and CD = 984 ± 144). Muscle fibre CSA was unchanged across all stimulation frequencies (*F*(3,14) = 1.511, *P =* 0.255; Figure 1d), while CDA (the area of tissue supplied by an individual capillary) was decreased by pacing at 10 Hz (717 ± 54 μm^2^) versus control (977 ± 148 μm^2^, *P =* 0.05; Figure 5e). Interestingly, heterogeneity of CDA was maintained following all stimulation regimes (*F*(3,14) = 1.356, *P =* 0.297; Figure 5f), consistent with CDA frequency distribution (Figure 1g). Remodelling of CD, CDA and logSD (Figure 5c–g) promoted a significant improvement in calculated tissue $P_{O_{2}}$ for muscles stimulated at 10 Hz (18.3 ± 1.0 mmHg vs. 13.5 ± 2.2 mmHg, *P =* 0.035), but 4 Hz (13.9 ± 3.5 mmHg, *P =* 0.994) and 40 Hz (18.0 ± 1.7 mmHg, *P =* 0.070) did not significantly alter $P_{O_{2}}$ profile (Figure 5h). Despite a significant ANOVA for modelled tissue hypoxia (*F*(3,14) = 3.419, *P =* 0.047), there was no significant change to the calculated extent of muscle hypoxia follow *post hoc* analysis for the 4 Hz (*P =* 0.533; Figure 5i), 10 Hz (*P =* 0.597; Figure 5i) or 40 Hz (*P =* 0.636; Figure 5i) ES groups.

#### 3.1.2 Tibialis anterior glycolytic cortex

The glycolytic cortex of TA (Figure 6a) showed a significant angiogenic response to ES (Figure 6b,c). While C:F only significantly increased at 4 Hz (2.63 ± 0.44, *P =* 0.003; Figure 6c) and 10 Hz (2.56 ± 0.51, *P =* 0.004; Figure 6b) compared to control (1.47 ± 0.09), CD significantly increased in response to 4 Hz (921 ± 159 mm^−2^, *P =* 0.005, Figure 6c), 10 Hz (1120 ± 176 mm^−2^, *P* < 0.0001, Figure 6c) and 40 Hz (829 ± 60 mm^−2^, *P =* 0.041, Figure 6c) stimulation compared to control (533 ± 105 mm^−2^), all becoming similar to that of the TA core (Figure 6b,c). The significant elevation in CD for 10 and 40 Hz is in part due to reduced fibre CSA, with decreases of 19% (*P =* 0.063) and 9% (*P =* 0.591), respectively. Cumulatively, these changes in capillary number and CSA lead to a significant reduction in CDA across all three pacing regimes (*F*(3,14) = 19.78, *P* < 0.0001; Figure 6e). Interestingly, heterogeneity in CDA was decreased following pacing at 4 Hz (*P =* 0.028; Figure 6f,g), with no significant changes seen in the 10 and 40 Hz stimulated groups (Figure 6f,g). Estimated tissue $P_{O_{2}}$ was more than doubled in all groups when compared to control (5.47 ± 1.74 mmHg, *F*(3,14) = 11.41, *P* < 0.0001, Figure 6h), while there was a significant decrease in the extent of predicted tissue hypoxia from 0.15% to <0.01% for all pacing strategies employed (*F*(3,14) = 25.442, *P* < 0.0001; Figure 6i).

#### 3.1.3 Compartmental effects of electrical pacing

To quantify heterogeneity within the TA in response to pacing, compartmental ratios (core:cortex) were calculated (Figure 7a). Cumulative changes in C:F and CD across the compartments indicate that the lower frequency stimulation parameters preferentially increased capillary content in the glycolytic cortex (Figure 7b,c) with 4 Hz (normalized C:F = 0.84 ± 0.23, *P =* 0.017; normalized CD = 1.13 ± 0.24, *P =* 0.003) and 10 Hz pacing (normalized C:F = 0.95 ± 0.17, *P =* 0.099; normalized CD = 1.23 ± 0.16, *P =* 0.01), compared to control. By contrast, despite 40 Hz pacing evoking a significant angiogenic response in the cortex (Figure 6c), normalized C:F (1.06 ± 0.18, *P =* 0.437) and CD (1.58 ± 0.20, *P =* 0.385) did not differ from control tissue when the TA was considered as a whole. Similar changes in fibre size across the core (Figure 5d) and cortex (Figure 6d) in response to ES produced an equivalent normalized CSA in all groups (*F*(3,14) = 0.574, *P =* 0.642; Figure 7d). Interestingly, significant decreases in CDA across the core (Figure 5e) and cortex (Figure 6e) following ES produced a graded response after normalization: 4 Hz increased CDA by 75% (*P =* 0.002) and 10 Hz by 54% (*P =* 0.025), further supporting the preferential adaptive remodelling of glycolytic tissue, while the increase in CDA after 40 Hz in both the core (Figure 5e) and cortex (Figure 6e) only partially elevated the normalized CDA (22%, *P =* 0.594). Heterogeneity of capillary supply (logSD) was not significantly affected by pacing (*F*(3,14) = 1.643, *P =* 0.225, Figure 7f), although normalized tissue $P_{O_{2}}$ was significantly decreased by all stimulation regimes (*F*(3,14) = 9.051, *P =* 0.001, Figure 7g).

#### 3.1.4 Muscle function

The EDL fatiguability displayed a frequency‐dependent response, with pacing at 10 and 40 Hz significantly improving FI by 58% (0.75 ± 0.13, *P =* 0.004; Figure 7h) and 73% (0.82 ± 0.15, *P =* 0.001; Figure 3h), respectively, while the 23% increase in FI at 4 Hz (0.58 ± 0.07, *P =* 0.448) was not statistically different from control (0.47 ± 0.06, Figure 7h). Measurement of hindlimb blood flow showed that the peak blood flow was similar, irrespective of pacing strategy (*F*(3,17) = 0.405, *P* = 0.751; Figure 7i), highlighting that the functional adaptations to stimulation were independent of changes in arterial blood supply.

### 3.2 Metabolomics analysis

#### 3.2.1 Multivariate analysis

**FIGURE 8:**
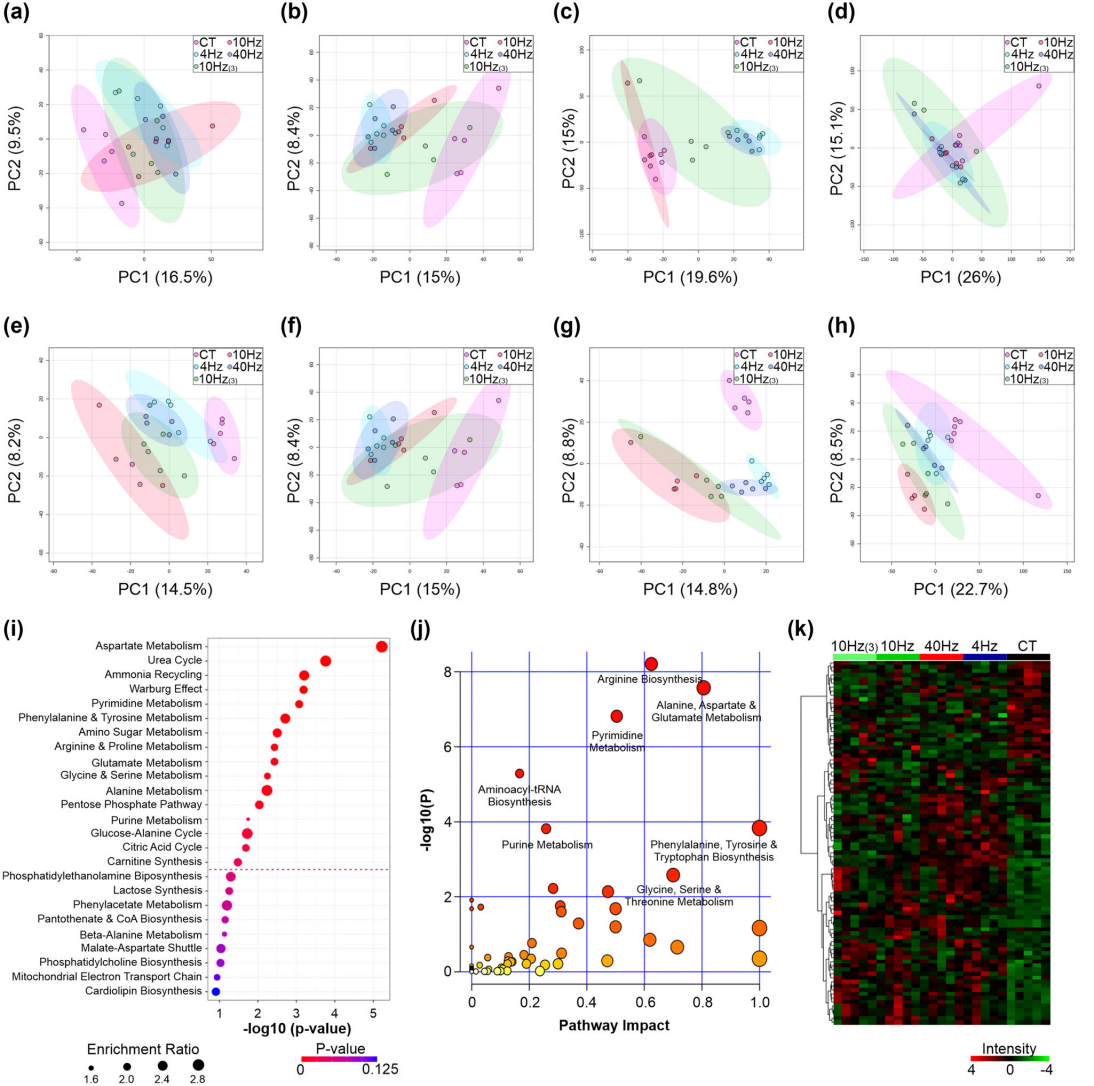
Multivariate analysis for all ion features detected by mass spectrometry. (a–h) Principal component analysis (a–d) and partial least squares‐discriminant analysis (e–h) for ion features detected by different methods; ion exchange (a, e), C18‐reverse phase (b,f), derivatized‐C18 (c,g) and hydrophobic interaction liquid chromatography (HILIC) (d,h). Data represent direct comparison if experimental data with modelled data (*Q* ^2^ and *R* ^2^) and permutation analysis to quantify the integrity of the data. Elliptical areas represent 95% confidence intervals. (i–k) Metabolite enrichment analysis (i) and pathway impact analysis (j) and for all identified compounds, with heatmap showing relative intensity of all significantly different identified compounds (k). Dashed pink line presents cut‐off for *P =* 0.05.

**FIGURE 9:**
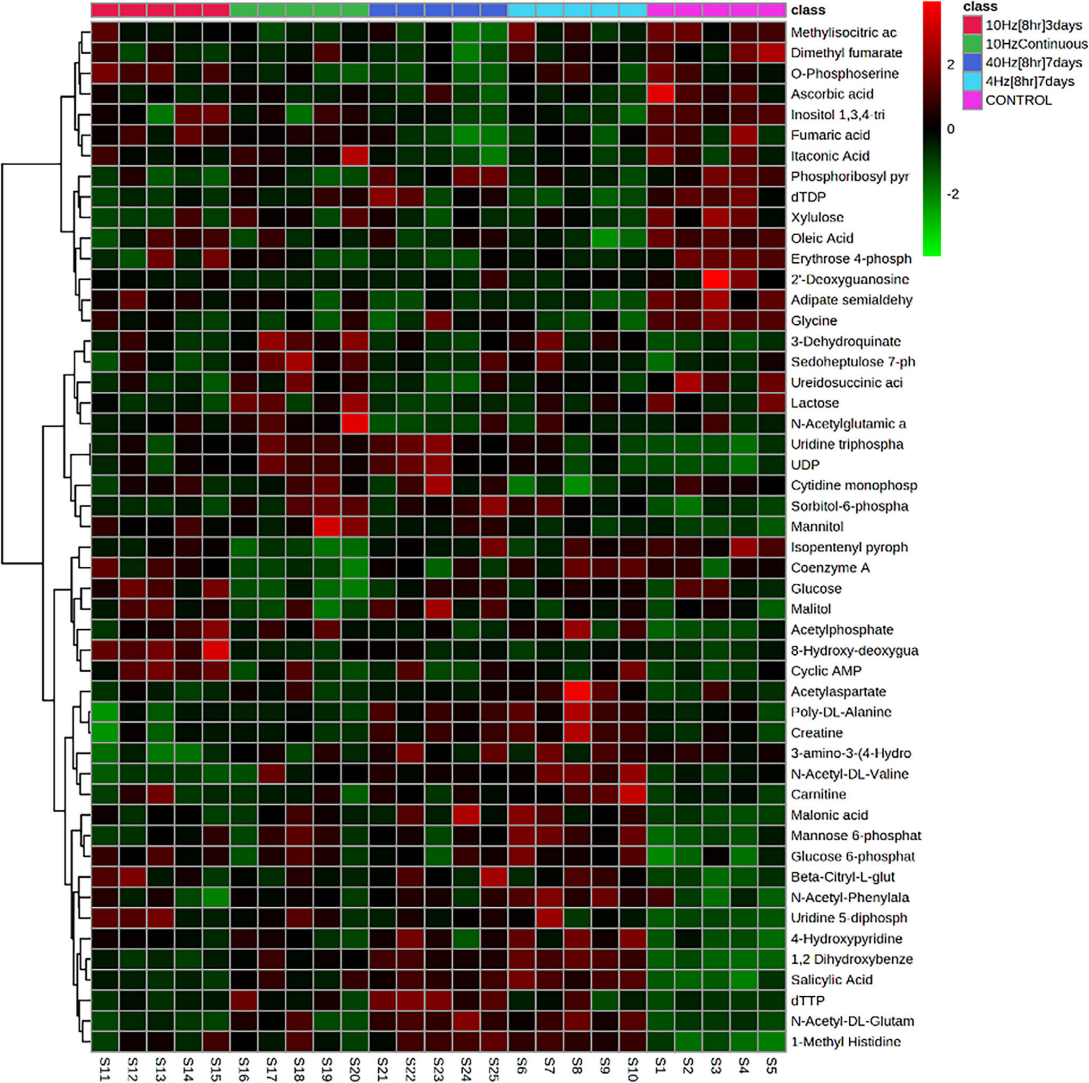
Metabolomics heatmap. Heatmap to show the effect of pacing strategies on statistically significant metabolites following single factor ANOVA (FDR threshold <20%; statistical significance *P* < 0.05). *n* = 5 samples/group.

Multivariate analysis of all four chromatographic methods using PCA (Figure 8a–d) and PLS‐DA (Figure 8e–h) indicated poor separation between experimental groups. For ion exchange, chromatography modelling showed that data were poorly resolved by PLS‐DA (*R* ^2^ = 0.999; *Q* ^2^ = 0.474; Figure 8e), and permutation analysis (*P =* 0.94; Figure 8e) showed that the modelled data was overfitted, and hence interpretations were unreliable; FDR was estimated as 0.001. For C18 reversed phase analysis the modelled effect of the experimental data poorly represented the observations (*R* ^2^ = 0.999; *Q* ^2^ = 0.470; Figure 8f) and the degree of separation between experimental groups reflected some ‘overfitting’ of the data when assessed by permutation analysis (*P =* 0.08; FDR was estimated as 0.006). For amino acid analysis (Figure 8g) the experimental data also reflected an ‘overfitted’ dataset when assessed using permutation analysis (*P =* 0.13, Figure 8g), showing poor correlation with the modelled data; FDR was estimated as 0.001. For HILIC analysis the experimental data poorly reflected the modelled data (*R* ^2^ = 0.999; *Q* ^2^ = 0.451; Figure 8h; FDR was estimated as 0.010), although permutation analysis indicated that the data were modelled adequately to reflect the measured changes (*P =* 0.04; Figure 8h). Hence, more focused analyses were required (Figure 9).

#### 3.2.2 Enrichment analysis

For the 240 identified compounds, metabolite enrichment (Figure 8i) and pathway impact analysis (Figure 8j) was performed using MetaboAnalyst4.0 and established that amino acid metabolism pathways were primarily affected by ES of skeletal muscle, with aspartate metabolism showing the greatest enrichment (15 of 16 metabolites – data not shown). In addition, pathway impact indicated that arginine biosynthesis and alanine, aspartate and glutamate metabolism were impacted the most strongly, demonstrating that ES led to modulation of amino acid metabolism. Of the identified compounds, analysis of the relative intensity by single‐factor ANOVA indicated a group of 102 metabolites (Figure 8k) significantly different from control samples.

#### 3.2.3 Carnitine synthesis

**FIGURE 10:**
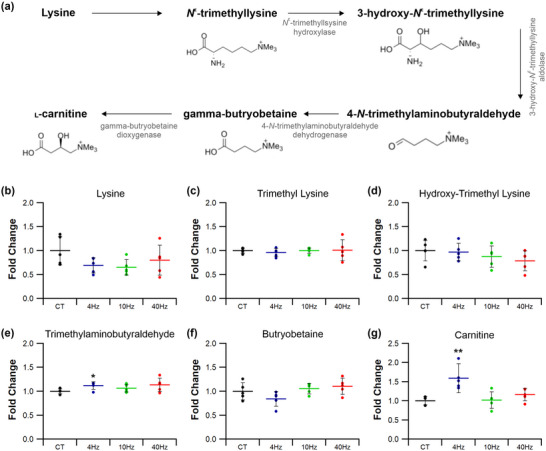
Effects of electrical pacing on metabolites present in the synthesis of carnitine in tibialis anterior muscle. (a) Identified compounds present in the metabolic pathway associated with carnitine synthesis. (b–f) ES, independent of stimulation frequency, did not significantly alter lysine (b), trimethyllysine (c), hydroxy‐trimethyllysine (d), trimethylaminobutyraldehyde (e) or butryobetaine (f) levels. (g) However, 4 Hz stimulation did significantly enhance muscle carnitine. Statistical significance represented as: *P < 0.05, **P < 0.01 vs. control tissue (CT) (*n* = 5 per group).

**FIGURE 11:**
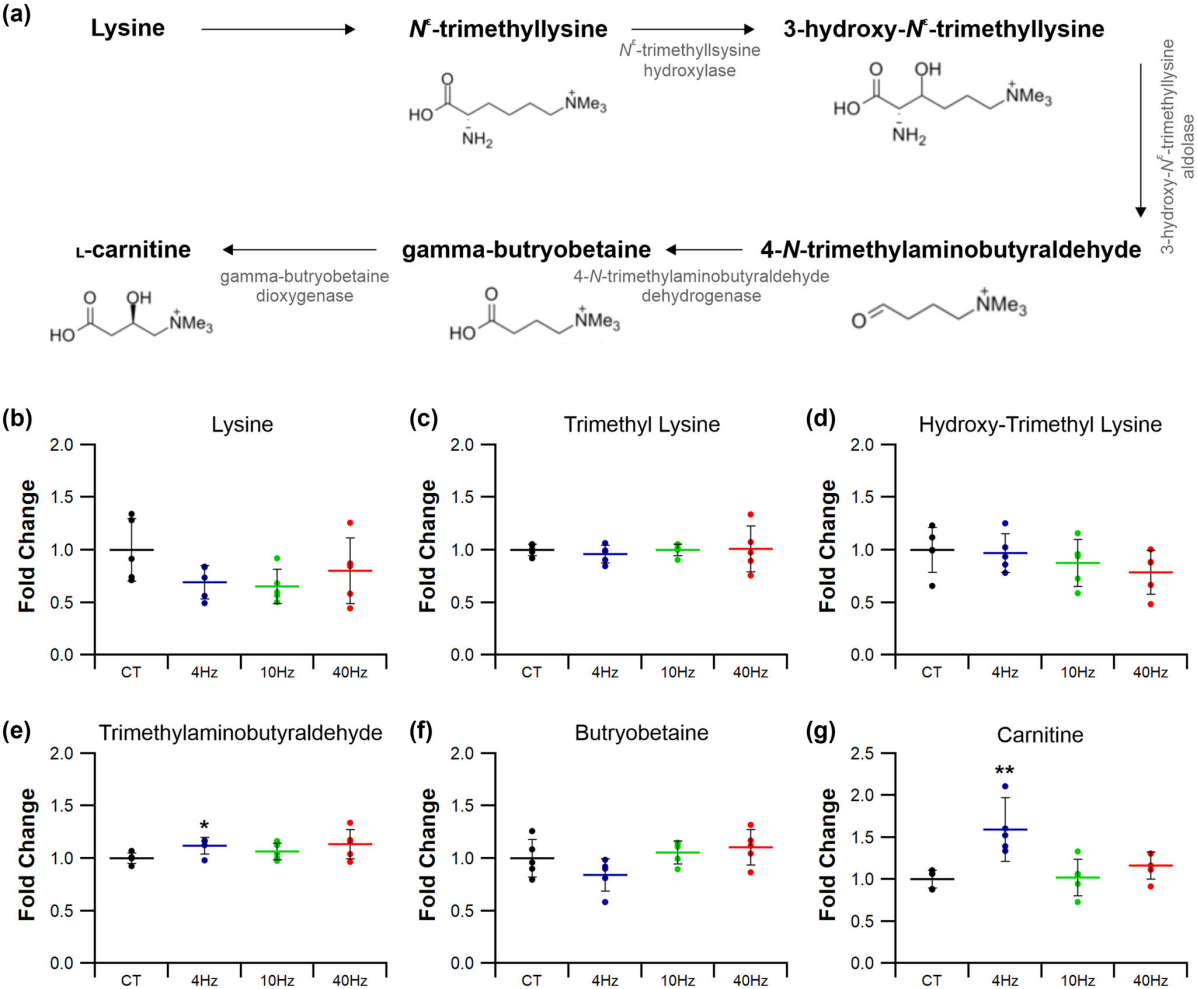
Temporal response of 10 Hz pacing on metabolites present in the synthesis of carnitine in tibialis anterior muscle. (a) Identified compounds present in the metabolic pathway associated with carnitine synthesis. (b) Lysine was reduced after just 3 days of ES but no longer significant after 7 days of ES. (c–g) Neither 3 nor 7 days of 10 Hz stimulation influenced trimethyllysine (c), hydroxy‐trimethyllysine (d), trimethylaminobutyraldehyde (e), butryobetaine (f) or carnitine (g) levels. Statistical significance represented as: *P < 0.05 vs. control tissue (CT) (*n* = 5 per group).

Carnitine is intimately coupled with oxidative metabolism of fatty acids and facilitates transport of acyl‐residues into mitochondria. Identified metabolites were mapped onto the pathways associated with carnitine synthesis, where ES induced significant alterations (Figures 10 and 11). Whilst 10 Hz_(3)_ pacing decreased muscle lysine levels by 50% (*P =* 0.022; Figure 11b), other stimulation regimes had no effect on tissue lysine levels (4 Hz *P =* 0.666; 40 Hz *P =* 0.489; Figure 10b). Both trimethyl lysine (TML) (Figure 10c; 4 Hz *P =* 0.274; 10 Hz *P =* 0.525; 40 Hz *P =* 0.763) and hydroxy‐trimethyl lysine (HTML – Figure 10d; 4 Hz *P =* 0.539; 10 Hz *P =* 0.722; 40 Hz *P =* 0.621) were unaffected by any pacing strategy. However, trimethylaminobutyraldehyde (TMABA) was increased by 20% following pacing at 4 Hz (*P =* 0.039; Figure 10e) although other stimulation regimes had no effect on TMABA levels (10 Hz *P =* 0.926; 40 Hz *P =* 0.568; Figure 10e). Muscle carnitine levels were increased by 50% following stimulation at 4 Hz (*P =* 0.033; Figure 10g), but were unchanged for all other ES regimes (10 Hz *P =* 0.802; 40 Hz *P =* 0.094; Figure 10g).

#### 3.2.4 Histidine metabolism

**FIGURE 12:**
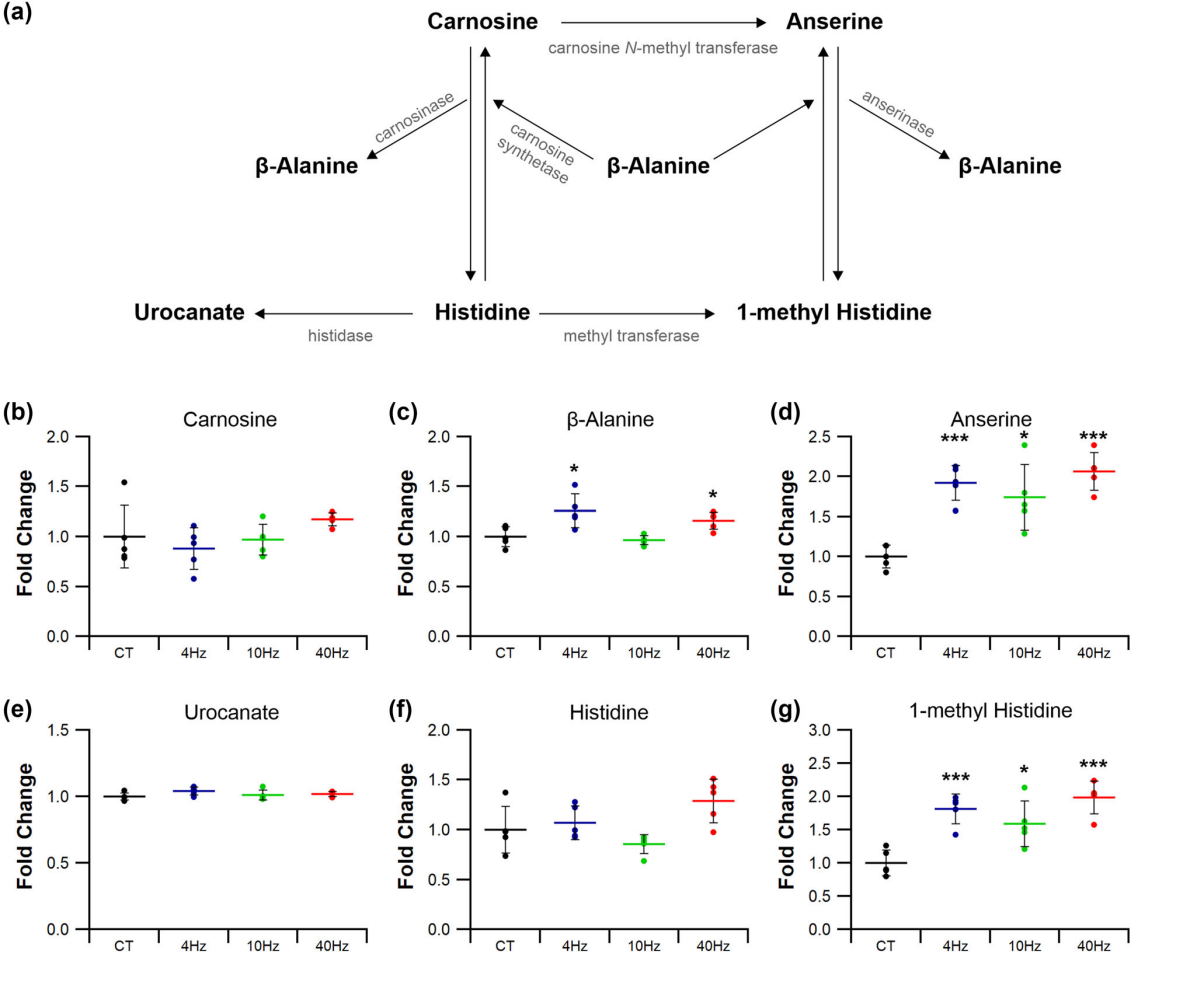
Effects of electrical pacing on metabolites present in the metabolism of histidine and 1‐methyl histidine in tibialis anterior muscle. (a) Identified compounds present in the metabolic pathway associated with the metabolism of histidine. (b–d) There were no significant changes in carnosine (b) in response to ES; however, there was an enhancement in β‐alanine (c) and anserine (d). (e–g) Urocanate (e) and histidine (f) levels were unaffected by 7 days of ES, while 1‐methyl histidine (g) was significantly elevated in response to ES. Statistical significance represented as: *P < 0.05, **P < 0.01, ***P < 0.001 vs. control tissue (CT) (*n* = 5 per group).

**FIGURE 13:**
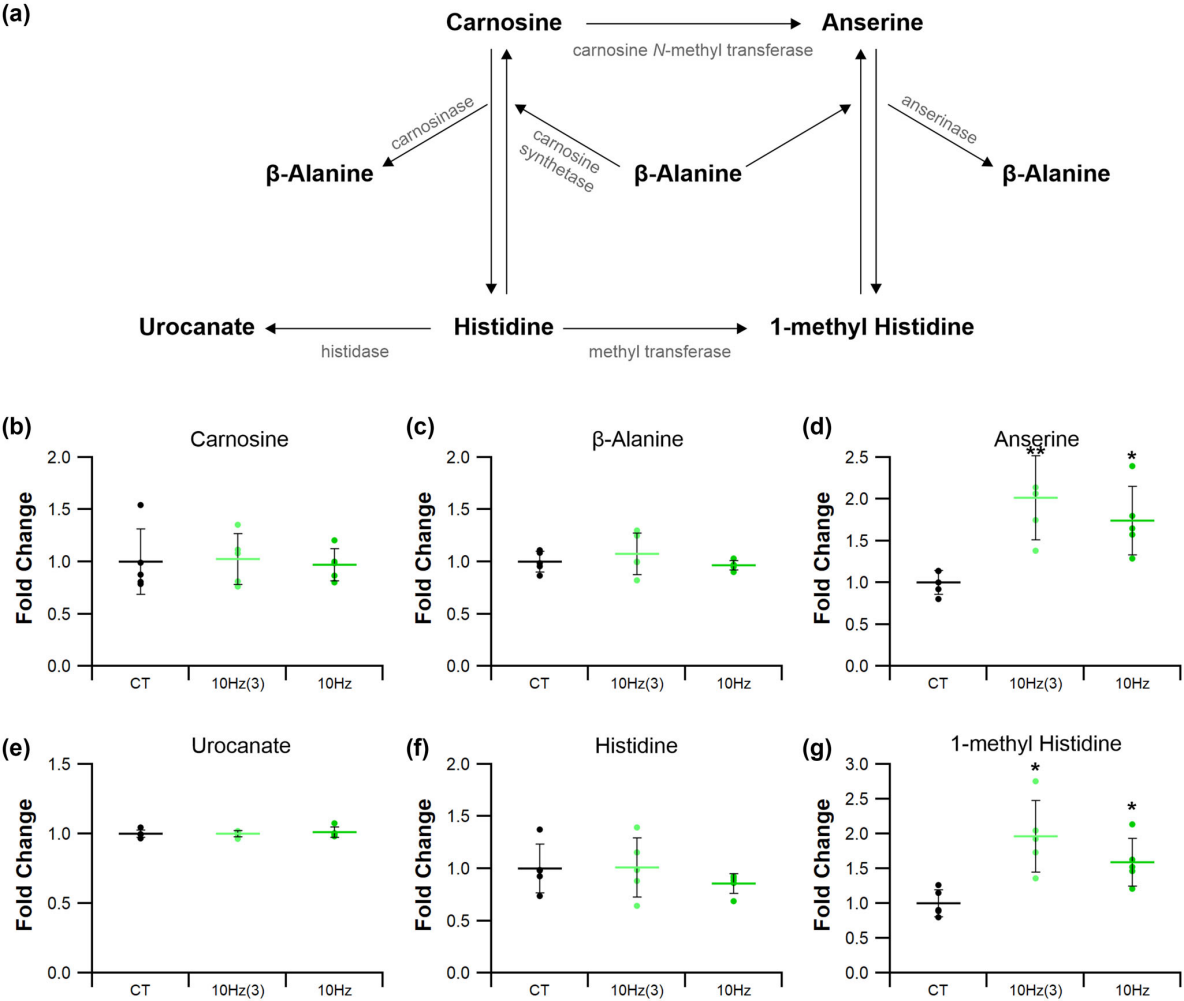
Temporal response of 10 Hz pacing on histidine and 1‐methyl histidine metabolites in tibialis anterior muscle. (a) Identified compounds present in the metabolic pathway associated with histidine. (b–d) 10 Hz stimulation for 3 or 7 days had no significant effect on carnosine (b) or β‐alanine (c) levels. (d) Anserine was significantly elevated after just 3 days of ES and was still significantly elevated after 7 days. (e–g) Urocanate (e) and histidine (f) remained unchanged after 10 Hz at both time points, while 1‐methyl histidine (g) was significantly elevated after just 3 days of stimulation, maintaining significance after 7 days. Statistical significance represented as: *P < 0.05 vs. control tissue (CT) (*n* = 5 per group).

Indicators of histidine metabolism in muscle reflects amino acid turnover (Figures 12a and 13a). Tissue levels of carnosine (4 Hz *P =* 0.455; 10 Hz *P =* 0.466; 40 Hz *P =* 0.679), urocanate (4 Hz *P =* 0.101; 10 Hz *P =* 0.549; 40 Hz *P =* 0.061) and histidine (4 Hz *P =* 0.691; 10 Hz *P =* 0.315; 40 Hz *P =* 0.846) (Figure 12b, e and f, respectively) were all unaffected by stimulation, irrespective of the ES regime adopted. Tissue β‐alanine levels were increased by 25% after 4 and 40 Hz stimulation (4 Hz *P =* 0.0301; 40 Hz *P =* 0.021; Figure 12c), whereas muscle paced at 10 Hz_(3)_ or 10 Hz was not significantly different from control (10 Hz_(3)_ *P =* 0.112; 10 Hz *P =* 0.157; Figures 12c and 13c). Stimulation led to a 2‐fold increase in anserine levels for 4 and 40 Hz (4 Hz *P* < 0.0001; 40 Hz *P* < 0.0001; *P =* 0.007 for 10 Hz_(3)_ and *P =* 0.015 10 Hz, Figures 12d and 13d). Additionally, tissue 1‐methyl histidine levels (Figure 12g) were approximately doubled by all ES regimes compared with control (4 Hz *P* < 0.0001 and 40 Hz *P =* 0.00015; Figures 12g and 13g: for 10 Hz_(3)_ *P =* 0.0089 and 10 Hz *P =* 0.016).

#### 3.2.5 Kynurenine metabolism

**FIGURE 14:**
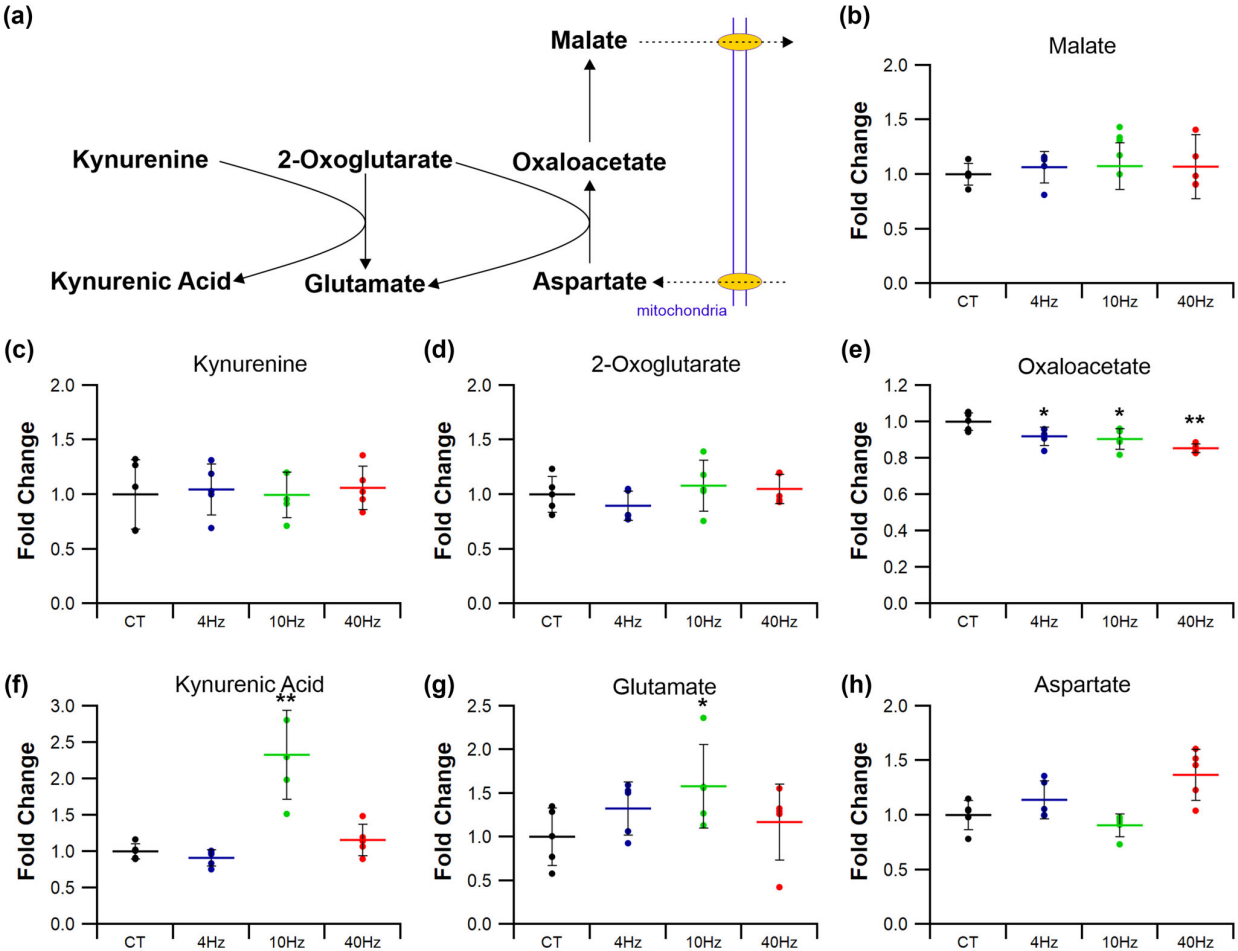
Effects of electrical pacing on metabolites present in the metabolism of kynurenine and kynurenic acid in tibialis anterior muscle. (a) Identified compounds present in the metabolic pathway associated with the metabolism of kynurenine. (b–e) Malate (b), kynurenine (c) and 2‐oxoglutarate (d) were unaffected by 7 days of ES, while oxaloacetate (e) was significantly decreased in response to 4, 10 and 40 Hz stimulation. (f–h) 10 Hz ES significantly elevated kynurenic acid (f) and glutamate (g) levels, with aspartate (h) remaining unchanged across all ES groups. Statistical significance represented as: *P < 0.05, **P < 0.01 vs. control tissue (CT) (*n* = 5 per group).

**FIGURE 15:**
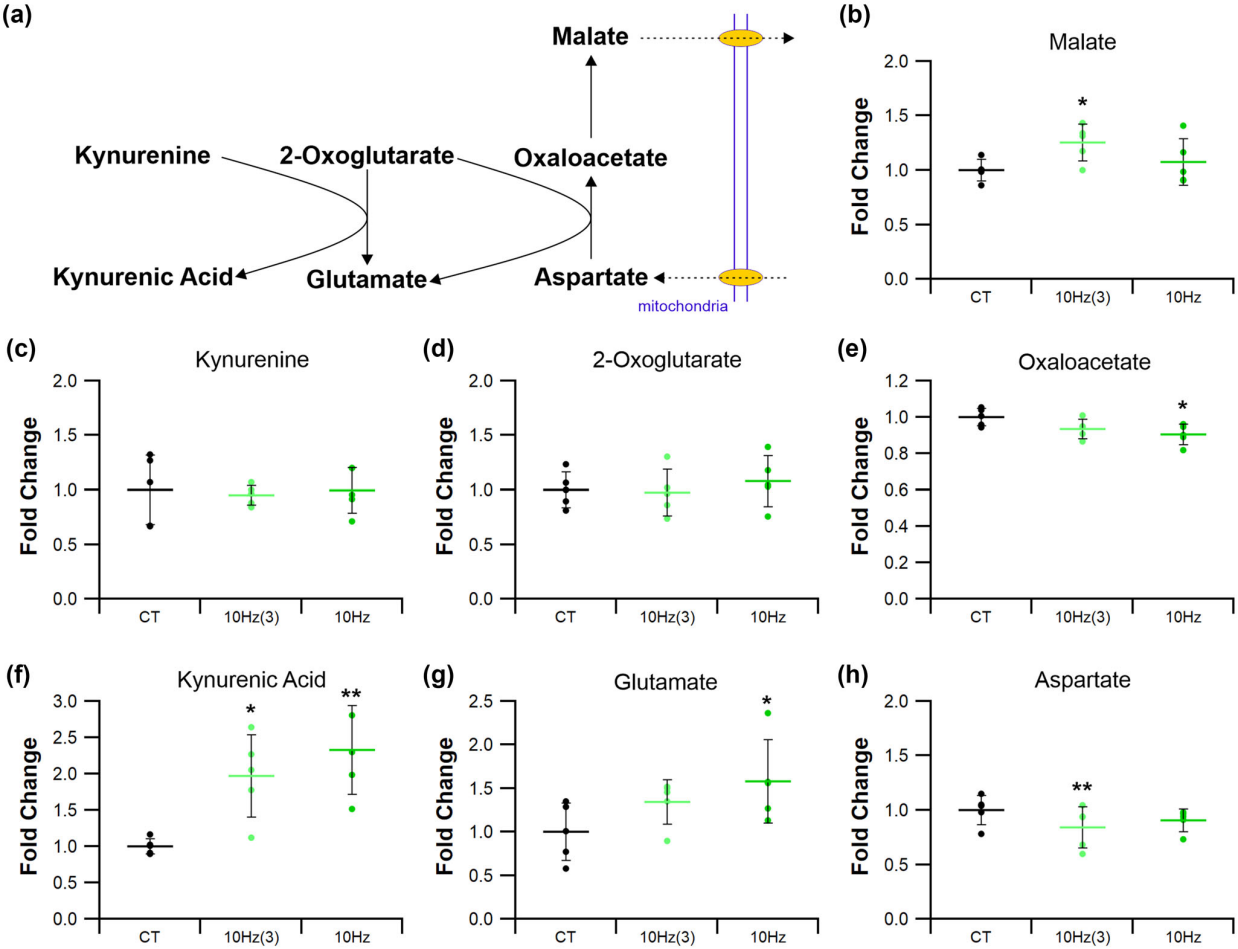
Temporal response of 10 Hz pacing on kynurenine and kynurenic acid metabolites in tibialis anterior muscle. (a) Identified compounds present in the metabolic pathway associated with kynurenine. (b) Malate levels were significantly elevated after just 3 days of ES, which was reduced after 7 days. (c,d) Kynurenine (c) and 2‐oxoglutarate (d) remained unchanged by 10 Hz stimulation across both time points. (e) Oxaloacetate decreased with just 3 days stimulation, becoming significantly reduced at 7 days. (f) 10 Hz ES significantly enhanced kynurenic acid levels after just 3 days and maintaining significance at 7 days. (g) Glutamate was unchanged after just 3 days becoming significantly elevated after 7. (h) Finally, aspartate was significantly reduced after 3 days of ES, recovering to control levels after 7 days. Statistical significance represented as: *P < 0.05, **P < 0.01 vs. control tissue (CT) (*n* = 5 per group).

Kynurenine metabolism is associated with the potential to augment the malate–aspartate shuttle (Agudelo et al., 2019), increasing electron transfer into mitochondria. This pathway (Figures 14 and 15) may be affected by ES, as 10 Hz_(3)_ increased muscle malate levels by 20% (*P =* 0.034; Figures 14b and 15b), but other pacing regimes had no effect (4 Hz *P =* 0.051; 10 Hz *P =* 0.063; 40 Hz *P =* 0.071; Figure 14b). Muscle kynurenine levels were unaffected by ES (4 Hz *P =* 0.806; 10 Hz_(3)_ *P =* 0.757; 10 Hz *P =* 0.982; 40 Hz *P =* 0.733; Figures 14c, 15c), although kynurenic acid levels were increased following both 10 Hz_(3)_ (*P =* 0.048; Figure 15f) and 10 Hz (*P =* 0.003; Figure 14f) regimes. Muscle 2‐oxoglutarate levels were unchanged by ES compared with control (4 Hz *P =* 0.300; 10 Hz_(3)_ *P =* 0.853; 10 Hz *P =* 0.552; 40 Hz *P =* 0.602; Figures 14d, 15d), but 10 Hz increased muscle glutamate levels by 50% (*P =* 0.029; Figures 14g and 15g). ES also decreased muscle oxaloacetate levels by 20% following stimulation at 4 and 10 Hz (4 Hz *P =* 0.048; 10 Hz *P =* 0.034; Figure 14e), and 40 Hz by 25% (*P =* 0.009; Figure 14e), whilst the 10 Hz_(3)_ regime had no effect (*P =* 0.063, Figure 15e).

## 4 DISCUSSION

We have demonstrated that indirect ES of skeletal muscle is able to drive phenotypically targeted angiogenesis resulting in distinct functional adaptive remodelling, but that structural changes are not directly linked to those seen in the metabolome. The potency of 10 Hz pacing was highlighted by increases in both C:F and CD across the entire TA, the latter leading to a decreased CDA and consequent increase in modelled muscle fibre $P_{O_{2}}$, which underpins improved muscle endurance capacity. Plasticity of the glycolytic compartment is particularly noteworthy in this regard, showing significantly increased capillarity and likely oxygenation in response to all pacing regimes, while the more oxidative core only responded to higher frequency stimulation. By contrast, our metabolomic analysis suggested that metabolite levels from pathways typically associated with endurance (glycolysis, TCA cycle, β‐oxidation) were largely unaffected by pacing strategy, although anabolic pathways were upregulated (carnitine, histidine, kynurenine metabolism). It is unlikely that structural remodelling is complete after just 7 days, indicating either biochemical adaptation is a second order effect or that prior metabolic capacity is sufficient to accommodate the requirements of augmented muscle activity, further evidenced by elevated methyl histidine levels following pacing (Bouletreau et al., 1987; Iwatsu et al., 2017).

Histological analysis confirmed that within the glycolytic cortex fibres were larger, with a lower capillary supply than those in the oxidative core, which consequently had a higher calculated tissue $P_{O_{2}}$ (Al‐Shammari et al., 2019; Deveci & Egginton, 2002; Kissane et al., 2019). This structural profile characterizes phenotypically distinct structural adaptive remodelling, in combination with shifts in metabolism, supporting the differential requirements for oxidative *vs*. glycolytic metabolism (Annex et al., 1998) that facilitate endurance versus acute intensive activity. Interestingly, glycolytic fibres demonstrated greater adaptative response to ES, approximately doubling CD and consequently halving the capillary supply area and enhancing peripheral oxygen delivery; this response is not matched in the oxidative compartment of TA. Our data also show a functional correlate, as ES improved muscle fatigue resistance. Similar findings have been reported previously (Egginton & Hudlicka, 1999; Scott et al., 1985), where arterial blood flow was unaffected by stimulation, indicating that improvements in EDL function were driven by intrinsic changes within the muscle tissue. Functional improvement in endurance with ES coincided with the increased capillary content/reduced CDA, and consequent reduction in oxygen diffusion distances from capillary to metabolically active fibres predicted to enhance fatigue resistance (Al‐Shammari et al., 2014; Kissane et al., 2020; Tickle et al., 2020), providing a clear link between the pacing‐derived remodelling of the microcirculation and improved muscle performance. Interestingly, we note an apparent mismatch between functionally relevant structural adaptations and changes to enabling metabolism, as quantified by metabolomic analysis. This is consistent with other studies showing fatigue resistance of electrically paced muscle peaking at 14 days, whilst oxidative capacity (estimated as citrate synthase activity) took longer to reach a maximum (Simoneau et al., 1993). However, metabolic profiling based on a few readouts is likely to be unrepresentative of the network of inter‐related pathways involved; a more holistic view requires unbiased outputs.

We infer from the metabolomic analysis that the diverse pacing regimes employed produced similar overall effects on the ion features detected by all chromatography methods, leading to an inability to statistically discriminate between groups. It may be that stimulation directly affected only a relatively small number of metabolic pathways, or that the pacing regimes did not exceed the capacity of the metabolic pathways examined to support increased muscle activity. The latter is unlikely, given the duration of continuous imposed activity, and that pacing significantly enhanced the FI with higher frequency regimes (10 and 40 Hz), suggesting that functional adaptation had indeed occurred. However, the relative contribution from changes to metabolic profile and enhanced substrate delivery due to increased capillary supply remains unclear. For example, increased muscle activity as a result of ES increased expression of genes contributing to energy metabolism via glycolysis and ATP synthases, in addition to myofibrillar enhancement (myosin heavy chains 1 and 2A; Gondin et al., 2011), and is associated with increased levels of VEGF mRNA that facilitated an increase in CD (Annex et al., 1998; Hang et al., 1995); thus, supply and demand may be co‐regulated (Yan et al., 2011).

However, enrichment and pathway impact analysis indicated that the principal pathways involved in adaptation to pacing were associated with amino acid biosynthesis and the urea cycle, suggesting that turnover of amino acids and anabolism are critical to the adaptation phase. Increased abundance of methyl histidine, anserine and β‐alanine may also support progressive remodelling of skeletal muscle as a consequence of pacing, although use of methyl histidine as a marker for muscle amino acid turnover may be controversial (Harris, 1981). While multiple organ systems can contribute to systemic levels of methyl histidine, the majority is present in muscle (Millward & Bates, 1983; Wassner & Li, 1982) but with a very slow turnover compared with the gastrointestinal tract (Calles‐Escandon et al., 1984), and urinary excretion was increased in both rats and humans following exercise (Dohm et al., 1982). More recent human experiments suggests that ES of skeletal muscle may decrease the loss of methyl histidine and metabolite turnover, preventing any reduced muscle performance post‐surgery (Bouletreau et al., 1987; Iwatsu et al., 2017). Our use of methyl histidine as an index of muscle turnover is therefore appropriate, as directly measuring muscle methyl histidine content rather than the urinary excretion is more closely linked to TA metabolism. That the response appears unaffected by ES suggests that remodelling is incomplete, and extending ES duration may restore methyl histidine and anserine to control levels.

The increase in TA carnitine levels for muscles paced at 4 Hz was unexpected. Given the obligate role that carnitine plays in metabolism of acylcarnitines, an increase in fatty acid β‐oxidation was anticipated to support a more aerobic phenotype. This may represent a transient adaptation in response to ES, as histological data showed little improvement in capillarity or $P_{O_{2}}$. However, ES may also affect the malate–aspartate shuttle, which plays an important role in translocation of reducing equivalents (NADH) into the mitochondria, with the potential to increase oxidative phosphorylation within the TA. In support of this was the potential increase in synthesis of kynurenic acid to facilitate translocation of electrons across the mitochondrial membrane. Recent investigations highlight the potential for kynurenine metabolism in muscle to support the malate–aspartate shuttle (Agudelo et al., 2019), and kynurenine aminotransferase is highly‐expressed in oxidative muscle fibres (Wyckelsma et al., 2020). Moreover, endurance exercise was associated with an increase in kynurenine aminotransferase expression (Allison et al., 2019; Schlittler et al., 2016). Together, these observations indicate that indirect ES may increase metabolic load on the TA muscle, and as a consequence metabolic adaptation may facilitate augmentation in electron transfer.

With the increasing interest in ES as a method to facilitate motor recovery across a range of debilitating pathologies (Jones et al., 2016), these data provide a novel insight into the acute remodelling potential of skeletal muscle. Our data highlight the differential structural response of skeletal muscle to altered frequencies of muscle stimulation, with the higher frequency of stimulation having a greater structural and functional adaptive response. Additionally, these data suggest that metabolomic readouts may not be the most appropriate indices of functional adaptation in a clinical model at acute time points. More work is required to establish the sub‐acute and chronic response of microvascular structure, metabolic status and muscle function to realize the therapeutic potential of this strategy.

In conclusion, we have demonstrated that indirect ES for 7 days at varying stimulation frequencies is capable of driving differential structural adaptation, which leads to improvements in muscle performance. Higher frequency stimulation regimes better improve capillarity across the whole muscle, increasing fatigue resistance, whereas lower frequency stimulation only improved capillary supply in the glycolytic cortex. Interestingly, improved fatigue resistance at 7 days appears to be mainly associated with structural adaptations, rather than changes to the muscle metabolome. However, following ES at 40 Hz, it did not show the greatest increase in CD, CDA or logSD, despite having the greatest enhancement in fatigue resistance, suggesting additional factors may indeed contribute. Finally, metabolic remodelling is unlikely to have plateaued at 7 days, and the subsequent influence of metabolism on function cannot be fully inferred without further study. This work underscores the versatility of indirect ES as a therapeutic strategy, demonstrating phenotypically distinct structural adaptations are possible through manipulation of activity patterns, and highlights the importance of local capillary distribution as a determinant of functional capacity: just having more capillaries is not necessarily beneficial.

## AUTHOR CONTRIBUTIONS

Experimental design: Roger W. P. Kissane, Stuart Egginton; animal surgery: Roger W. P. Kissane, Peter G. Tickle, Stuart Egginton; sample collection and histological analysis: Roger W. P. Kissane; metabolomics analysis: David Hauton; in situ muscle function and blood flow: Peter G. Tickle; manuscript draft: David Hauton. All authors have read and approved the final version of this manuscript and agree to be accountable for all aspects of the work in ensuring that questions related to the accuracy or integrity of any part of the work are appropriately investigated and resolved. All persons designated as authors qualify for authorship, and all those who qualify for authorship are listed.

## CONFLICT OF INTEREST

The authors declare no conflict of interests.

## Data Availability

All the raw data (capillary counts, oxygen transport modeller outputs, fatigue index and metabolomics) presented in this manuscript are available online at the University of Liverpool's Research Data Catalogue (https://doi.org/10.17638/datacat.liverpool.ac.uk/1991).
